# Loss of Parkinson Disease Protein 7 (PARK7) upregulates ROS and cell migration and is associated with recurrent pregnancy loss

**DOI:** 10.1186/s10020-025-01344-w

**Published:** 2025-12-05

**Authors:** Zhiqi Yang, Emily Hellwich, Nisha Mohd Rafiq, Alvin Joselin, Doo Soon Im, Gaurav Kaushik, Yogesh Singh, Biserka Mulac-Jericevic, Huanhuan Jiang, Irene Gonzalez-Menendez, Leticia Quintanilla-Martinez, Sara Y. Brucker, Tilman E. Schäffer, Madhuri S. Salker

**Affiliations:** 1 Department of Women’s Health, University Hospital Tübingen, Calwerstraße 7/6, 72076 Tübingen, Germany; 2 Institute of Applied Physics, University of Tübingen, Tübingen, Germany; 3 Interfakultäres Institut Für Biochemie, University of Tübingen, Tübingen, Germany; 4 Department of Clinical Neurosciences, Hotchkiss Brain Institute, Cumming School of Medicine, University of Calgary, Calgary, Canada; 5 Institute of Medical Genetics and Applied Genomics, University of Tübingen, Tübingen, Germany; 6 Department of Physiology and Immunology, Medical School, University of Rijeka, Rijeka, Croatia; 7 Reproductive Medicine Center, Department of Obstetrics and Gynecology, the First Affiliated Hospital of Anhui Medical University, Hefei, China; 8 Institute of Pathology and Neuropathology, Comprehensive Cancer Center, University Hospital Tübingen, Tübingen, Germany; 9 Core Facility Histology, Faculty of Medicine Tübingen, University Hospital Tübingen, Tübingen, Germany; 10 Cluster of Excellence iFIT (EXC2180) “Image-Guided and Functionally Instructed Tumor Therapies, University of Tübingen, Tübingen, Germany; 11 Department of Obstetrics and Gynecology, Faculty of Medicine, University of British Columbia, Vancouver, BC V5Z 4H4 Canada; 12 Women’s Health Research Institute, Vancouver, BC V6H 3N1, Canada; 13 BC Children’s Hospital Research Institute, Vancouver, British Columbia BC V5Z 4H4 Canada

**Keywords:** DJ-1, Decidualization, Recurrent pregnancy loss, Endometrium, Cytoskeleton, Palladin

## Abstract

**Background:**

Successful implantation is dependent on a synchronous dialogue between several proteins that act to control cellular dynamics including actin and microtubule reorganisation and cell motility. An impaired crosstalk can lead to complications including recurrent pregnancy loss (RPL) though the precise mechanisms remain unclear. Parkinson disease protein 7 (*PARK7*; encoding DJ-1), characterised for its participation in neurodegeneration, has emerged as a novel cytoskeletal and antioxidant regulator however, the role of DJ-1 in early pregnancy is unknown.

**Methods:**

We employed systems biology approaches and functional studies in both human and murine models to examine the expression and role of DJ-1 during the window of implantation. LC–MS/MS proteomics analysis was conducted to identify proteins with differential expression between decidualized endometrial stromal cells (EnSC) with and without DJ-1 knockdown. Further, DJ-1 expression was manipulated using loss and gain of function strategies to investigate its impact on reactive oxygen species (ROS), the actin cytoskeleton and cellular motility. Lastly, knockdown of Palladin (a key actin regulator) and overexpression of Glutathione peroxidase 3 (GPX3) were used to investigate the downstream effects.

**Results:**

Endometrial DJ-1 had the highest expression during the implantation window and loss of DJ-1 was associated with pregnancy loss in both humans and mice. The proteomics data revealed dysregulation of cytoskeletal protein, Palladin and antioxidant enzymes. Knockdown of DJ-1 using siRNA led to elevated ROS levels, increased actin polymerisation and resulted in increased cell motility towards aneuploidic signals. Conversely, DJ-1 overexpression led to the reversal of these effects. Moreover, knockdown of downstream target Palladin restored abnormal actin polymerization and prevented cell motility towards aneuploidic chemotactic signals. Further, overexpression of GPX3 mitigated ROS production and restored cell migration.

**Conclusions:**

Taken together, our findings identified an unexpected function of the DJ-1-Palladin axis in the endometrium and its function as a redox-sensitive chaperone and in cytoskeleton remodelling and migration. Thus, uncoupling of this axis may result in adverse pregnancy complications including recurrent pregnancy loss.

**Supplementary Information:**

The online version contains supplementary material available at 10.1186/s10020-025-01344-w.

## Background

Recurrent pregnancy loss (RPL) is estimated to affect approximately 5% of couples and is a common obstetric complication (Quenby et al. 2021; Genovese and McQueen 2023). It is characterized by the recurrence of two or more subsequent losses prior to 24 weeks of gestation (Dimitriadis et al. 2020). Various etiological factors have been implicated to account for these losses and are screened for anatomic, endocrine, immunologic, thrombophilic and genetic risk factors (Tong et al. 2024). Despite concerted investigative efforts, approximately 50% RPL cases lack a clearly defined etiology (Yu et al. 2023). Furthermore, as the number of euploid miscarriages increases the likelihood of a successful pregnancy diminishes (Ogasawara et al. 2000), implying a potential role of endometrial factors in driving RPL. Given that few interventions effectively increase live-birth rates in women affected by RPL (RPL EGGo et al. 2022), it is evident that the underlying mechanisms remain largely unclear (Lucas et al. 2020).

After ovulation, the increase in progesterone levels induces a temporary receptive state in the endometrium for embryo implantation to occur during the mid-luteal phase (Horne et al. 2000). This window marks the onset of extensive tissue remodelling known as decidualization, which is characterized by the biochemical and morphological reprogramming of endometrial stromal cells (EnSC) into decidual cells (Lucas et al. 2020). Decidualization is a multistep process starting with an acute cellular stress response, characterised by a surge in reactive oxygen species (ROS), the secretion of proinflammatory mediators (Erkenbrack et al. 2018; Salker et al. 2012) and modulation of actin and microtubule dynamics and cell migration towards the embryo (Ihnatovych et al. 2009). A key characteristic of decidual cells is their ability to respond to multiple stress signals, involving suppression of the c-Jun N-terminal kinase (JNK) and forkhead box O3 (FOXO3a) pathways, increased expression of stress defence proteins and ROS scavengers and the release of growth and angiogenic factors (insulin-like growth factor binding protein-1; IGFBP-1), which are necessary for the establishment of a conducive endometrial microenvironment (Christian et al. 2002; Maruyama and Yoshimura 2008; Gellersen and Brosens 2014; Leitao et al. 2010). However, the decidual process is highly susceptible to disruptions caused by various factors, including immune system dynamics, extracellular matrix (ECM) interactions, glucose metabolism, high ROS levels and hormonal fluctuations (Murata et al. 2022). Aberrant expression of these factors or processes can compromise decidualization and lead to a variety of common reproductive disorders, including implantation failure and RPL.

DJ-1 was initially characterized as an oncogene product involved in a Ras-related signal transduction pathway (Nagakubo et al. 1997). In humans, DJ-1 is encoded by the PARK7 gene, which has been identified as causing autosomal recessive early onset Parkinson’s disease (PD) (Bonifati et al. 2003). Interestingly, the DJ-1 gene spans 23.86 kilobases (kb) on chromosome 1 which comprises of 7 exons interspersed with 17 distinct GT-AG introns splice sites (Thierry-Mieg and Thierry-Mieg 2006), thus the possibility of having a greater chance of alternative splicing at the gene locus. Furthermore, DJ-1, is a part of the large, multiclade DJ-1/ThiJ/PfpI superfamily, which are evolutionarily conserved from bacteria to *Homo sapiens* (Buneeva and Medvedev 2021; Smith and Wilson 2017). Structurally, DJ-1 monomers adopt a helix-fold-helix flavodoxin-like fold, characterized by 11 β-strands (β1–β11) and 8 α-helices (αA–αH), and functions as a homodimer based on biochemical and structural analyses (Honbou et al. 2003). DJ-1 functions as a multifaceted protein with both antioxidant and transcriptional activities (Kahle et al. 2009). Its involvement extends to critical cellular functions in other processes, such as the regulation of mitochondrial homeostasis and cell proliferation (Lazzari et al. 2021). In addition to its associations with various cancers and PD, studies have indicated that an aberrant level of DJ-1 also plays a prominent role in the pathogenesis of multiple disorders encompassing, type 2 diabetes, inflammatory diseases and in immune cell development and maintenance of their cellular physiological functions including pH and ROS levels (Dolgacheva et al. 2019; Eberhard and Lammert 2017; Kawate et al. 2017; Sun et al. 2020; Zhang et al. 2020; Singh et al. 2015, 2020; Zhou et al. 2017). However, the role of DJ-1 in early pregnancy remains scant. As of current knowledge, the relationship between DJ-1 and human endometrial decidualization remains unknown.

Despite these emerging insights, the mechanism by which the maternal decidua develops the molecular profile needed to support rapid expansion during early gestation remains unclear. Accumulating evidence has posited the association between impaired decidualization and heightened susceptibility to subsequent pregnancy loss (Salker et al. 2010). Importantly, decidualized EnSC from RPL patients fail to discriminate the presence of (low-quality) C-grade embryos and exhibit abnormal migratory behaviour toward these embryos compared with control patients (inhibited migration) (Weimar et al. 2012). The failure to differentiate between poor- and high-quality embryos can be considered as a lack of quality control- commonly known as the “Selection Failure” hypothesis (Weimar et al. 2012; Quenby et al. 2002; Aplin et al. 1996). However, the precise mechanistic underpinnings governing this relationship remain largely obscure. We hypothesized that the loss of DJ-1 in the endometrium increases ROS levels, deregulates the ECM protein milieu and augments abnormal cell migration in women suffering from RPL. In this study, we present evidence that DJ-1 expression increases during decidualization in both humans and mice. Furthermore, we observe a correlation between the loss of DJ-1 and RPL in both humans and mice. Importantly, our findings indicate that DJ-1 levels influence decidualization by modulating ROS levels and Palladin expression, consequently impacting on cell morphology, actin polymerization and cell migration towards chemotactic signals derived from aneuploidic trophoblast cells. Additionally, these effects were reversed with DJ-1 overexpression and abnormal ROS levels are mitigated in the presence of an overexpressing Glutathione peroxidase 3 (GPX3) plasmid. These insights underscore a novel role for DJ-1 in both endometrial decidualization, cytoskeletal regulation and the pathogenesis of RPL.

## Methods

### Patient selection and sample collection

The study was approved by the Anhui Medical University research ethic committee (No. PJ2018-07–20). The number of samples were determined and approved using a Power calculation (G*Power, with an 80% effect size). The endometrial samples were collected by the Reproductive Medicine Center of the First Affiliated Hospital of Anhui Medical University. Patients had not received hormone or steroidal treatment 3 months prior to sample collection (Tables 1 and 2). Controls were women without a history of RPL. Written informed consent was obtained from all participants in accordance with The Declaration of Helsinki 2000 guidelines. Endometrial samples were obtained at surgery and snap frozen immediately in liquid nitrogen before storage at − 80 °C for later downstream analysis.

**Table 1:** Patient demographics and characteristics (Fig. 1g) RNA-seq (GSE65102)

	**Age (years)**	**Live Birth**	**Number of Losses**	**BMI (kg/m**^2^)	**LH + Day (days)**
Controls	36.6 ± 1.36	0	0	24.6 ± 1.42	7.2 ± 0.13
RPL	37.5 ± 0.79	0.3 ± 0.15	4.9 ± 0.52	25.44 ± 1.02	7.9 ± 0.45

Data shown are arithmetic mean ± SEM (*n* = 10 in each group); Controls; unexplained = 5; male factor = 2; tubal disease = 1; endometriosis = 1; polycystic ovary syndrome = 1; *RPL* recurrent pregnancy loss, *BMI* body mass index; LH +: days after the luteinizing hormone peak

### Cell culture

Primary human EnSC from Applied Biological Materials Inc (#T0533, Abm, Canada), were maintained at 37 °C in a humidified 5% CO_2_ atmosphere in DMEM/F-12 medium (Gibco, United Kingdom) containing 10% (v/v) dextran coated charcoal (DCC) stripped (#C6197, Sigma, United States) fetal bovine serum (#A5256701, FBS, Gibco, Germany), 1% (v/v) antibiotic–antimycotic solution (#15240062, Gibco, United States), and 0.25% (v/v) L-glutamine (#25030081, Gibco, United Kingdom). Cells were passaged when the confluency reached 80%. Before treatment or transfection, the culture medium was changed to 2% (v/v) DCC stripped FBS, 1% (v/v) antibiotic–antimycotic solution and 0.25% (v/v) L-glutamine. The cells were decidualized with 0.5 μM 8-Bromo-cAMP (#1140, 8-Bromo-cAMP, Tocris, United Kingdom) and 1 μM Medroxyprogesterone 17-acetate (#M1629-1G, MPA, Sigma, Germany). All work was carried out in a Class I laminar flow hood. Cells were routinely tested for mycoplasma and always gave a negative result.

### Transfection

EnSC were plated in 6-well plates at a density of 200,000 cells per well in 2% DCC FBS media. For knockdown experiments, EnSC were transfected with *PARK7* small interference RNA (siDJ-1, 136368, Invitrogen, Germany) or *PALLD* siRNA (siPALLD, #126881, Invitrogen, Germany) by using Lipofectamine RNAiMAX Transfection Reagent (#13778075, Invitrogen, Germany) according to the manufacturer’s protocol. For overexpression experiments, pGW1-Myc-DJ1-WT was a gift from Mark Cookson (Addgene plasmid # 29347; http://n2t.net/addgene:29347; RRID: Addgene_29347) (Miller et al. 2003). The human *Glutathione Peroxidase 3* (*GPX3*, NM_002084) tagged ORF clone was obtained from Origene Technologies (SKU: RG207758). The ORF is cloned into the pCMV6-AC-GFP vector and was used for transient expression in mammalian cells. Transfection was performed by using the Lipofectamine™ LTX Reagent with PLUS™ Reagent (#15338100, Invitrogen, Germany) in accordance with the manufacturer's instructions. After 24-h the transfection mix was discarded and replaced with decidualization treatment medium. Cells were collected for downstream analysis.

### Total RNA extraction and real-time quantitative PCR (qRT-PCR)

Total RNA was extracted from cells using TRizolTM reagent (Invitrogen). 1 µg of RNA was utilized to synthesize cDNA using the ThermoScientific Maxima™ H Minus cDNA Synthesis Master Mix with dsDNase (#M1681, Invitrogen, Germany). mRNA concentration was measured by using a Nanodrop. qRT-PCR was performed on the QuantStudio 3 Real-Time PCR System (Invitrogen) by using sets of gene-specific primers and the PowerUp™ SYBR® Green Master Mix (#A25742, Invitrogen). The relative differences in PCR product amounts were quantified by the ^ΔΔ^CT method, using ribosomal L-19 as an internal control (Livak and Schmittgen 2001). Experiments were performed in triplicate. Melting curve analysis and agarose gel electrophoresis confirmed amplification specificity. All the gene-specific primers used in this study were designed using primeblast (NCBI) and purchased from Sigma (Ye et al. 2012).

### Protein extraction and G/F actin separation

To quantify actin polymerization in EnSC, these cells were incubated in 80 µL of Triton X-100 extraction buffer containing 0.3% Triton X-100 (#3051, Carl Roth, Germany), 5 mM Tris (#T1503, Sigma–Aldrich; pH 7.4), 2 mM EGTA (#E3889, Sigma–Aldrich), 300 mM sucrose (#4621, Carl Roth), 2 µM phalloidin (#P2141, Sigma–Aldrich), 1 mM PMSF, 10 µg mL − 1 leupeptin (#L8511, Sigma–Aldrich), 20 µg mL − 1 aprotinin (#A6279, Sigma–Aldrich), 1 mM sodium orthovanadate (#S-6508, Sigma–Aldrich), and 50 mM NaF (#S-1504, Sigma–Aldrich) for 10 min on ice (G-actin separation). The supernatant containing the soluble proteins was removed by aspiration. Cells were then incubated in 100µL of Radioimmunoprecipitation Assay (RIPA) buffer (ThermoFisher, #89901) for 10 min on ice. The Triton X-insoluble pellet (F-actin separation) was scraped from the plate. Any remaining insoluble material was removed by centrifugation. Equal volumes of each fraction were boiled in Laemlli Buffer protein loading buffer at 95 °C for 5 min.

### Protein extraction for whole cell or tissue lysates

Total whole cell lysates were prepared by lysing the adherently cultured EnSC in Laemmli buffer containing 0.5 M Tris hydrochloride (#9090.1, Roth, Germany) pH 6.8, 20% Sodium dodecyl sulfate (SDS, #L4509, Sigma, Germany), 0.1% Bromophenol blue (#15375.01, Serva, Germany),1% beta mercaptoethanol (#M6250, Sigma, Germany), and 20% glycerol (#3783.1, Roth, Germany). Whole cell protein lysates were heated at 95 °C for 5 min and either were stored at −20 ℃ or followed with Western blotting procedure below.

Human tissue samples were extracted using the previous method (Kawate et al. 2017). In brief, human endometrial biopsies were washed in phosphate-buffered saline (PBS) (#D8537, Sigma-Aldrich) to remove any external contaminants. Subsequently, the tissues were homogenized in RIPA buffer (#89901, ThermoFisher), supplemented with freshly added protease inhibitors (#05892970001, Roche), using the pestle method. Homogenization was carried out until a uniform lysate was obtained. The homogenized samples were then centrifuged at 13,000 Relative Centrifugal Force (RCF) for 10 min at 4 °C to pellet cellular debris and other insoluble components. The resulting supernatant containing solubilized proteins was carefully collected and transferred to fresh tubes for further analysis. To determine the protein concentration in the extracted samples, the Bradford protein assay method was employed. Briefly, Bradford reagent (#B6916, Sigma-Aldrich) was added to the samples according to the manufacturer’s instructions. The absorbance of the resulting solution was measured at the appropriate wavelength using a spectrophotometer, and protein concentrations were determined using a standard curve generated with known concentrations of Bovine Serum Albumin (BSA) (#10735086001, Roche).

### Western blotting

Extracts were loaded on to a 12% sodium dodecyl sulfate poly-acrylamide gel (SDS-PAGE) using the XCell SureLock® Mini-Cell apparatus (Invitrogen) followed by electrophoresis. The protein from the gel was transferred onto poly-vinylidenefluoride membrane (#15289894, Amersham Biosciences, Germany). After air drying the membranes were activated in 100% methanol and subsequently blocked using 5% non-fat milk or BSA for 1-2 h at room temperature (RT). Membranes were probed overnight at 4 °C with primary antibodies: human DJ-1 antibody (1:1000, #5933, Cell Signaling), human Palladin antibody (1:1000, #8518, Cell Signaling), human pan-actin antibody (1:1000, #12748, Cell Signaling), human GAPDH (1:1000, #2118L, Cell Signaling) was used for loading control. After washing 3 times with TBS-T each for 10 min, the membranes were incubated with HRP-conjugated anti-rabbit secondary antibody (1:2000, #7074 s, Cell Signaling) at RT for 1 h, followed by washing with 3 times TBS-T. Protein bands were detected using a chemiluminescent detection kit (WesternBright™ ECL, #34580, ThermoFisher, Germany) and visualized by using the iBright™ Imaging System (Invitrogen). Bands were quantified with ImageJ Software (Gallo-Oller et al. 2018). Whole-cell lysate samples were normalised to GAPDH and then the fold change was calculated relative to the control, except for human tissue samples, which were normalised to pan-actin and then the fold change was calculated relative to a single control patient. The original, uncropped Western blot images are provided in the Supplementary Information.

### Immunofluorescence

EnSC were plated on 12 mm round coverslips at a density of 3000 cells per coverslip. Treatment for decidualization was performed as described above. Post treatment the cells were fixed for 15 min with 4% paraformaldehyde (PFA), washed 3 times with PBS, and permeabilized for 10 min in 0.1% Triton X-100/PBS. The coverslips were blocked with 5% BSA in 0.1% TritonX-100/PBS for 1 h at RT and were probed overnight at 4 °C with primary antibody: human DJ-1 antibody (1:100, #5933, Cell Signaling), Palladin antibody (1:80, #NBP1-25959, NOVUS). After washing 3 times, coverslips were probed overnight at 4 °C with secondary antibody: Alexa Fluor 488 (4 µg/mL, #A-11008, Invitrogen) and Alexa Fluor 568 (2 µg/ml, #A-11004, Invitrogen). Cells were stained for F-actin with Phalloidin eFluor 660 (1:1000, # 50–6559-05, Invitrogen) for 30 min at room temperature. The coverslips were mounted with ProLong Gold antifade reagent with DAPI (#P36931, Invitrogen) on slides. Microscopy was performed with LSM 800 confocal laser scanning microscope (Zeiss) and EVOS M7000 cell imaging system (ThermoFisher) with 40X objective. Scale bar was 75 μm. Image analysis was performed using ImageJ (Khoa and Dou 2020). For each cell, the nucleus was identified based on DAPI staining, the regions of interest corresponding to the nucleus, cytoplasm and entire cell were manually outlined. Fluorescence intensity was then measured separately in different compartments. The nuclear cytoplasmic ratio was calculated as nuclear fluorescent intensity divided by cytoplasmic fluorescent intensity.

### Flow cytometry

EnSC were fixed with 4% PFA for 10 min and then permeabilized with 1X permeabilization buffer (#00–8333-56, eBioscience, Germany) and subsequently stained with 1 mg/mL of Deoxyribonuclease I, Alexa Fluor 488 (50 mg/mL, #D12371, ThermoFisher, Germany) for detection of G-actin, 1.0 µL of 1X fluorescent Phalloidin-eFluor 660 (#50–6559-05, eBioscience, ThermoFisher, Germany) for detection of F-actin. The abundance of the respective labels was measured using green (FITC) and red channel (APC) on FACSCanto II clinical flow cytometry system (BD Biosciences, Germany). Analysis was performed using Flowjo software (Flowjo LLC, USA). G- and F-actin geometric mean values were determined from the respective fluorescence and the ratio of G/F calculated from the geometric mean values.

EnSC were treated as described above. The percentage of viable, apoptotic and necrotic cells was estimated by flow cytometry using the AnnexinV apoptosis detection kit FITC (#88–8005-72, eBioscience, Germany) in accordance with the manufacturer's instructions. Briefly, cells were collected by trypsin and washed with PBS and 1X binding buffer, respectively, then cells were suspended in 1X binding buffer containing Annexin V-FITC solution (1:50 dilution). After that, cells were incubated at RT for 15 min, protected from light, and washed with 1X binding buffer again. After adding Propidium Iodide (PI) solution (#P4864, 1:100 dilution, Sigma, Germany), cells were incubated at RT in the dark for 10 min prior to flow cytometry for cell apoptosis analysis. Data was analysed by Flowjo software.

### Mass spectrometry (LC–MS/MS)

For proteome analyses, cell suspensions were prepared from decidualized EnSC with and without siDJ-1 transfection as mentioned above. Cells were lysed by adding lysis buffer [5% 1 M Tris/HCl pH 7,4, 2% 5 M NaCl, 1% Triton X 100, 1% PMSF, 4% protease inhibitor cocktail (Sigma-Aldrich, St. Louis, USA)] on ice. Ten micrograms of each sample were digested in solution with trypsin. After desalting using C18 stage tips, extracted peptides were separated on an Easy-nLC 1200 system coupled to a Q Exactive HFX mass spectrometer (ThermoFisher Scientific, Germany). The peptide mixtures were separated using a 90 min segmented gradient from to 10–33-50–90% of HPLC solvent B (80% acetonitrile in 0.1% formic acid) in HPLC solvent A (0.1% formic acid) at a flow rate of 200 nL/min. The 12 most intense precursor ions were sequentially fragmented in each scan cycle using higher energy collisional dissociation (HCD) fragmentation. Acquired MS spectra were processed with the MaxQuant software package (version 1.6.7.0) with the integrated Andromeda search engine (Cox and Mann 2008). Database search was performed against a target-decoy Homo sapiens database obtained from Uniprot, containing 103.859 protein entries and 286 commonly observed contaminants. Peptide, protein and modification site identifications were reported at a false discovery rate (FDR) of 0.01, estimated by the target/decoy approach. The LFQ (Label-Free Quantification) algorithm was enabled, as well as match between runs and LFQ protein intensities were used for relative protein quantification. Data analysis was performed using Perseus and STRING v11 database (Tyanova et al. 2016). The mass spectrometry proteomics data have been deposited to the ProteomeXchange Consortium via the PRIDE (Perez-Riverol et al. 2022) partner repository with the dataset identifier PXD051694.

### Glutathione peroxidase activity assay

GPX activity was measured using a commercial assay kit (#MAK437, Sigma, Germany) according to the manufacturer’s protocol. The assay is based on the GPX-catalyzed oxidation of reduced glutathione (GSH) to oxidized glutathione (GSSG). The generated GSSG is subsequently reduced back to GSH by glutathione reductase in the presence of nicotinamide adenine dinucleotide phosphate (NADPH). The rate of NADPH consumption, which is stoichiometrically linked to GPX activity, was monitored by measuring the decrease in absorbance at 340 nm.

### Wound healing assay

EnSC were seeded in six-well plate (100,000 cells per well). After 6 days of treatment until the cells were reaching 90–100% confluency, cells were synchronized by double thymidine (2 mM; #T1895, Sigma, Germany) blocking. The wells were then scratched with a 100 µL tip. Pictures were taken immediately (0 h) using the EVOS M7000 cell imaging system with a 4X objective (Olympus, Germany). Pictures were taken again after 24-h. For experiments simulating an aneuploid environment, supernatant from BeWo cells was collected and applied to the treated EnSC during the 24-h wound healing period. Assessment of wound healing was quantified using the ImageJ software.

### AFM force mapping

Atomic Force Microscope (AFM) experiments were performed on live EnSC with a commercial AFM setup (MFP3D Bio, Asylum Research, Santa Barbara, USA). EnSC were seeded on Petri dishes (#351006, Falcon) at a density of 3 × 10^4^ cells/cm^2^ and were treated as described above. Single EnSC were imaged using the force mapping mode. Maps of 10 × 10 force-vs-indentation curves were recorded on a 10 × 10 µm^2^ scan area on the central region of the cell using a pyramidal tip (Bio-MLCT, cantilever C, spring constant 0.01 N/m, side angle 35 ± 2°, Bruker AXS S.A.S., Champs-sur-Marne, France), which was calibrated with the thermal noise method (Cook et al 2006), and fitted with the pyramidal Sneddon model to determine the Young’s modulus, E (Bilodeau 1992). Additionally, the whole cell body was imaged by performing maps of 50 × 50 force-vs-indentation curves on a 90 × 90 µm^2^ scan area. The median of all Young´s modulus values in one map on a cell was considered representative of that cell. To minimize the influence of the underlying substrate, only stiffness values for cell regions with a height above 1 μm were considered and an indentation depth much smaller than the cell height was used.

### Cell viability assay

EnSC were seeded in 96-well plate (3000 cells per well) and treated as described above. After 6 days treatment, cells were incubated with 5 µL of CellTiter 96 Aqueous One Solution (#G3580, Promega, Germany) reagent for 3–4 h at 37°C. Absorbance at 490 nm for each well was measured on Varioskan™ LUX multimode microplate reader (#VLBL0TD2, ThermoFisher, Germany). The OD values were normalized to the control. Data are represented as percentage.

### BrdU assay

Cell proliferation was assessed using the Bromodeoxyuridine (BrdU) Cell Proliferation Assay Kit (QIA58, Sigma, Germany), according to the manufacturer’s protocol. Briefly, EnSC were seeded in 96-well plates and allowed to adhere overnight. BrdU labeling solution was added to each well and incubated for 2–24 h, depending on the proliferation rate of the cell line. After incubation, cells were fixed and DNA was denatured to allow BrdU detection. A peroxidase-conjugated anti-BrdU monoclonal antibody was applied, followed by a substrate solution to develop color. Absorbance was measured at 450 nm.

### Measurement of cellular ROS using DCFDA

EnSC were seeded in 96-well plate (3000 cells per well). To determine ROS production in decidualized EnSC with or without DJ-1 knockdown, cells were co-incubated with 10 μM 2′,7′ − dichlorofluorescin diacetate (DCFDA; #D6883, Sigma, Germany) for 30 min in the dark at RT. Cellular ROS production was measured at excitation and emission wavelengths of 499 and 522 nm, respectively, using the Varioskan™ LUX multimode microplate reader (#VLBL0TD2, ThermoFisher, Germany). ROS levels were expressed as relative fluorescence units (RFU). All measurements were performed in triplicate (Kim and Xue 2020).

### Animal and tissue collection

#### Dj-1 mating model

The generation and genotype of the DJ-1-deficient mice have previously been described in detail (Joselin et al. 2012). All animal procedures were approved by the University of Calgary Animal Care Committee (ACC Certification AC22-0072). Animals were maintained in strict accordance with the guidelines for the Use and Treatment of Animals set forth by the Animal Care Council of Canada and endorsed by the Canadian Institutes of Health Research. In brief, Wild-type (WT) females (10–12 weeks old) were placed with fertile males and vice versa, to ensure all the conceptuses were heterozygous, and the day a vaginal plug was observed was considered day 0.5 of pregnancy (dpc). The animals were euthanised by cervical dislocation at 7.5–9.5 dpc or until pups were delivered. Our study exclusively examined female mice because the disease modelled is only relevant in females.

#### Mouse implantation studies in wild type mice

Mice (BALB/c) were housed in animal facilities at the University of Rijeka, Faculty of Medicine and maintained in accordance with institutional and international guidelines. All experimental and surgical procedures involving mice complied with the Guide of Care and Use of Laboratory Animals and were approved by the University of Rijeka, Faculty of Medicine’s ethical committee. Genetically modified BALB/c, PRKO, and PRAKO mice were generously provided by John P. Lydon and Orla M. Conneely (Baylor College of Medicine Houston, Texas, USA). WT females (10–12 weeks old) were placed with fertile males, and the day a vaginal plug was observed was considered 0.5 dpc. The animals were euthanized by cervical dislocation at 2.5, 4.5, 7.5, and 9.5 dpc. Intravenous injection of 1% (w/v) Chicago sky blue dye (Sigma-Aldrich, St. Louis, MO) (0.1 mL/mouse) was used to visualize implantation sites. One uterine horn was fixed in 4% (w/v) PFA. Our study exclusively examined female mice because the disease modelled is only relevant in females.

### Immunofluorescence of mouse tissues

Tissue sections were deparaffinized and rehydrated through an ethanol gradient. Heat-induced antigen retrieval was performed in 10 mM sodium citrate, pH 6.0. To block nonspecific binding, tissue sections were incubated with 1% BSA in PBS containing 0.001% NaN_3_ for 1 h at RT. Tissue sections were incubated with primary antibodies diluted in 1% BSA in PBS containing 0.001% NaN_3_ overnight at 4° C in a humid environment.

For DJ-1 expression antibodies (D29E5) XP® Rabbit mAb (1:1000, #59335, Cell Signaling Technology), followed by Fluor 594 donkey anti rabbit IgG, 2 mg/mL, (#A21207, dilution 1:500, Invitrogen) were used. For COX2 expression goat polyclonal COX2 antibodies (1:100, #ab23672, Abcam, Cambridge. UK), followed by biotinylated horse anti-goat IgG (1:300, #BA-9500, Vector Labs,) were used. COX2 expression was visualizes by streptavidin (Alexa Fluor 488 nm Streptavidin, 1:1000 dilution, Molecular Probes, Life Technologies, Carlsbad, CA, USA).

Nuclei were visualized with 4’, 6-diamidino-2-phenylindole, dihydrochloride (DAPI, ThermoFisher Scientific, Rockford, IL, USA). The sections were mounted in Mowiol mounting media. Images were captured on Olympus imaging system BX51 equipped with DP71CCD camera (Olympus, Tokyo, Japan) and CellF imaging software was used. Images were edited using Photoshop CS6 (Adobe, San Jose, CA, USA).

### Histology

Uteri were fixed in 4% PFA and embedded in paraffin. For histology, 3–5 µm-thick sections were cut and stained with haematoxylin and eosin (H&E). Immunohistochemistry was performed on an automated immunostainer (Ventana Medical Systems, Inc.) according to the company’s protocols for open procedures with slight modifications. All slides were stained with the antibody Cleaved Caspase 3 (#ASP 175; Cell Signaling Technology). Appropriate positive and negative controls were used to confirm the adequacy of the staining.

For the microscopic analysis, the guidelines from the Histology Atlas of the Developing Mouse Placenta (Elmore et al. 2022) were used. Briefly, the placentas at the midsagittal point, given by the presence of the chorioallantoic attachment were selected and the appropriate specimens were chosen based on the developmental stage-matched. The histologic samples were then analyzed by an experienced pathologist (L. Quintanilla-Martinez). All samples were scanned with the Ventana DP200 (Roche, Switzerland) and processed with the Image Viewer MFC Application. Final image preparation was performed with Adobe Photoshop CS6.

### Data mining

In silico analysis was performed on the following publicly available datasets from the Gene Expression Omnibus (GEO): profiling gene expression during the peri-implantation phase and during implantation in the mouse uterus (GSE44451) (Xiao et al. 2014); midluteal endometrial biopsies from 10 women with RPL and 10 women with no history of losses (GSE65102) (Lucas et al. 2016). Bioinformatic analysis was performed on publicly available single cell sequencing data from the Single Cell Expression Atlas (Vento-Tormo et al. 2018).

### Statistical analysis and data visualisation

Data were analyzed by using the Student’s t-test or One-way analysis of variance (ANOVA) for significance in GraphPad Prism software (GraphPad software Inc., San Diego, CA, United States). Statistical data visualization was performed using box-and-whisker plots, violin plots, and bar graphs to illustrate the distribution and central tendency of experimental data. In box-and-whisker plots, the box spans the interquartile range (IQR), with the horizontal line indicating the median. Whiskers extend to the most extreme values within 1.5 × IQR from the lower and upper quartiles. Individual data points are overlaid to show sample distribution and variability. Violin plots combine a box plot with a kernel density estimate to display both summary statistics and the full distribution shape. The width of the violin reflects the probability density of the data, and the embedded box plot indicates the median and interquartile range. Individual data points are also shown. Bar graphs present the mean ± standard error of the mean (SEM), with individual data points overlaid to reflect biological variability. The number of independent biological experiments were denoted as, n. Statistical significance was considered when P value was less than 0.05. figures were made using GraphPad Prism 9 and Inkscape 1.4.2. Schematic images were created in BioRender. Salker M. (2025) https://BioRender.com/vghscgh**.**

## Results

### DJ-1 is expressed in the endometrium and deficiency of DJ-1 (PARK7 gene) is correlated with pregnancy loss in both humans and mice

To assess DJ-1 expression in normal human endometrial tissue, we initially conducted a bioinformatic analysis using publicly available single cell RNA-sequencing datasets (Vento-Tormo et al. 2018). We observed a high expression of *DJ-1* in the human decidua, especially within endometrial decidual cells (Supplemental Fig. S1a, b) (Vento-Tormo et al. 2018). Additionally, according to the Human Protein Atlas, DJ-1 is most abundantly expressed in the human stromal compartment and in the glands (Supplemental Fig. S1c) (Uhlen et al. 2005). Since the highest expression was seen in stromal cells all further downstream experiments were performed in EnSC.

**Fig. 1:**
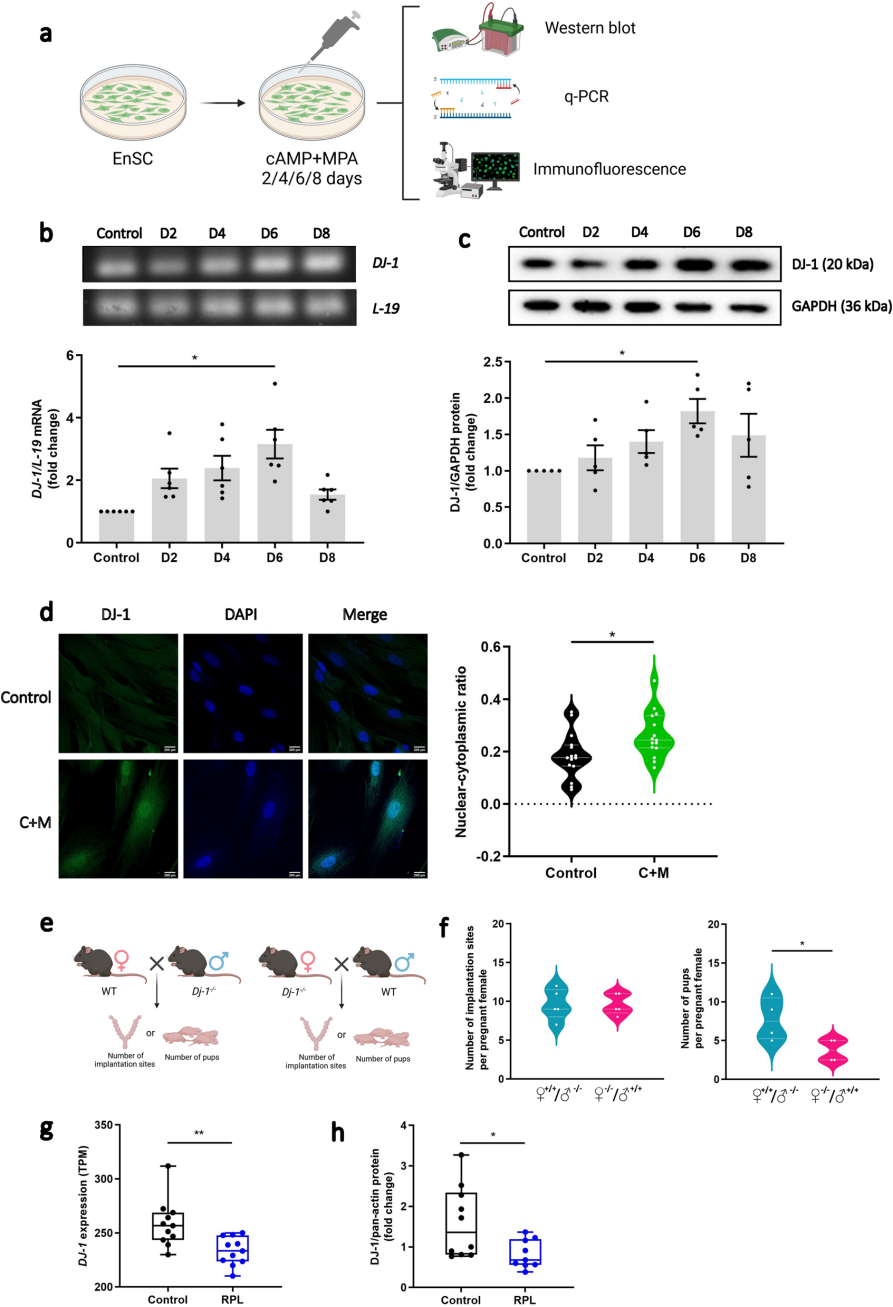
Effect of DJ-1 level on early pregnancy. **a** Schematic depiction of the approach of in vitro experiments. EnSC were treated by 0.5 μM 8-Br-cAMP and 1 μM MPA to induce in vitro decidualization up to 8 days (D) of treatment, followed by downstream experiments. Control EnSC remained untreated. **b ***DJ-1* mRNA levels in EnSC during decidualization were quantified by qRT-PCR (*n* = 6). Cycle threshold (Ct) values were normalized to the housekeeping gene, *L-19*. **c** Western blot analysis of DJ-1 expression during decidualization in EnSC (*n* = 5). GAPDH was used as a loading control. Data are presented as mean ± SEM. One-way ANOVA was used to calculate statistical significance between the groups *P < 0.05. **d** Immunofluorescence (IF) microscopy of untreated (upper panel) and decidualized (lower panel) EnSC showing DJ-1 subcellular localization. Quantification of DJ-1 nuclear-cytoplasmic fluorescence intensity ratio is shown (right). DJ-1: Alexa Fluor 488 (green); nucleus: DAPI (blue). Quantification performed from 3 experiments with 15 cells quantified for each condition. Scale bar = 200 µm. **e** Schematic depiction of mice mating model. **f** Number of pups per litter based on the breeding of *Dj-1*^−/−^ female mice crossed with WT males, or WT females crossed with *Dj-1*^−/−^ males (*n* = 4). Violin shows data distribution *via* kernel density, with median and interquartile range (IQR) indicated by embedded box plot. **g** Human *DJ-1* gene expression in endometrium of patients with Control and RPL (*n* = 10) (GSE65102). **h** Western blot analysis of DJ-1 protein expression in endometrial tissue from patients with controls (*n* = 10) and RPL (*n* = 9). Pan-actin was used as a loading control. Box represents the IQR, line indicates the median, whiskers show 1.5 × IQR. Unpaired t-test was used to calculate statistical significance. *P < 0.05, **P < 0.01

Next, to validate the expression of DJ-1 during decidualization, EnSC were subjected to treatment with cAMP and MPA (synthetic progestin) (C + M) every 48 h for a total of 8 days. Subsequently, samples were collected for downstream analysis (Fig. 1a). qRT-PCR analysis indicated a significant upregulation of *DJ-1* mRNA expression on the 6th day with a fold change of 3.16 following decidualization treatment (*P* = 0.0269; Fig. 1b), which was then reduced at day 8 representing the closure of the implantation window. We further observe that *DJ-1* transcripts are induced upon cAMP treatment (*P* = 0.0497; Supplemental Fig. S1d) after 6 days. Western blotting results demonstrated a significant increase in DJ-1 protein expression on the 6th day with a fold change of 1.75 (*P* = 0.0364; Fig. 1c and Supplemental Information). To further explore protein localization, immunofluorescence analysis was conducted to validate the expression and subcellular localization of DJ-1 in EnSC. As depicted in Fig. 1d and Supplemental Fig. S1e, DJ-1 was dispersed in both the cytoplasm and nucleus of untreated EnSC. The intensity of DJ-1 was significantly elevated in decidualized cells, particularly within the nucleus (*P* = 0.0209) indicating nuclear shuttling.

In view of its multifunctional role, Dj-1 could also be a putative regulator of murine pregnancy. We initially analysed publicly available microarray dataset (GSE44451) profiling gene expression during the peri-implantation phase (3.5 days post coitus; dpc) and during implantation (4.5 dpc) in the mouse uterus (Xiao et al. 2014). As depicted in Supplemental Fig. S1f, the expression of *Dj-1* exhibited an increase (*P* = 0.0440) during early pregnancy in mice. Consistent with previous findings at day 4.5 of pregnancy there was strong COX2 staining surrounding the murine implantation site, which was used as a positive control (Sucurovic et al. 2020, 2017). We employed double labelling and immunofluorescence techniques to compare murine Dj-1 and Cox2 (as a positive control) expression at day 4.5 of pregnancy. Our findings demonstrate the localization of Dj-1 surrounding the implantation site and distally from the implantation site in the glandular epithelium, primarily within the nucleus (Supplemental Fig. S1g). Given previous indications implicating impaired decidualization and susceptibility to subsequent pregnancy failure (Salker et al. 2010), our observations indicate that Dj-1 is essential for ongoing decidualization and pregnancy, and thus, the loss of Dj-1 will have a determinantal effect on pregnancy outcomes. To evaluate this conjecture, we counted implantation sites and litter sizes in *Dj-1*^−/−^ female mice mated with wild-type (WT) males and WT females mated with *Dj-1*^−/−^ males, thus eliminating the influence of the offspring’s genotype (Fig. 1e). In *Dj-1*^−/−^ females, the count of implantation sites at 8.5 dpc remained unchanged, however the average number of pups per litter dropped significantly, suggesting over 50% spontaneous fetal loss (P = 0.0422; Fig. 1f). H&E staining was performed to explore the structure of the implantation sites. As shown in Supplementary Fig. 1 g, at 8.5 dpc in the decidua from WT females crossed with *Dj-1*^−/−^ males, the villi are correctly formed as per the gestational age with abundant blood vessels (Supplemental Fig. S1h). However, in the placentas of *Dj-1*^−/−^ females crossed with WT males, the decidual cells are disorganized and the villi formation was delayed. At 9.5 dpc, a similar delay was observed in *Dj-1*^−/−^ females. In the placenta of WT females, the villi were fully established, while in the placentas of *Dj-1*^−/−^ females, the villi were thicker and fewer blood vessels were observed (Supplemental Fig. S1h). Furthermore, at Embryonic day (E) 8.5 the placentas of *Dj-1*^−/−^ females showed a slight increase of the apoptosis (Caspase 3 staining) when compared with the placentas of WT female (Supplemental Fig. S1i).

Given the abundant DJ-1 expression in the human endometrium and that the loss of murine matenal Dj-1 resulted in losses, we postulated whether DJ-1 expression was also altered in women with RPL. We examined published GEO transcriptome data from midluteal endometrial biopsies from 10 women with RPL and 10 women with no history of losses (GSE65102) (Lucas et al. 2016). The clinical cohort 1 (Table 1) had no difference in age or BMI. Women with RPL had 4.9 ± 0.52 more losses than the control group. As depicted in Fig. 1g and Table 1, there was a significant reduction in endometrial *DJ-1* mRNA transcripts (*P* = 0.0037). To confirm this, protein samples of human endometrial tissues were collected from an independent cohort of 9 women with RPL and 10 women with no history of losses (independent validation cohort, Table 2). In the second cohort, again there was no statistical difference between ages and BMI. There was a difference of 3.78 ± 1.22 losses (*P* = 0.0105) seen in the RPL group. As illustrated in Fig. 1h, Supplementary Information & Table 2, DJ-1 protein expression in the RPL group was significantly reduced (52% reduction) compared with the control group (*P* = 0.0253). The original blot images are provided in the Supplementary Information. Band intensities were quantified relative to pan-actin.

**Table 2:** Patient demographics and characteristics used in Western blots (Fig. 1h)

	**Age (years)**	**Live Birth**	**Number of Losses**	**BMI (kg/m**^2^)	**LH + Day (days)**
Controls	35.2 ± 2.70	0	0	22.4 ± 2.10	9 ± 0.70
RPL	33.12 ± 3.28	0	3.78 ± 1.22	21.4 ± 3.10	6.9 ± 1.30

Data shown are arithmetic mean ± SEM; Controls (those not experiencing pregnancy losses) = 10 RPL; Recurrent Pregnancy Loss = 9; BMI; Body Mass Index; LH +; days after the luteinizing hormone peak

### Loss of DJ-1 disrupts decidualization with decreased expression of decidual markers through the regulation of Palladin

**Fig. 2:**
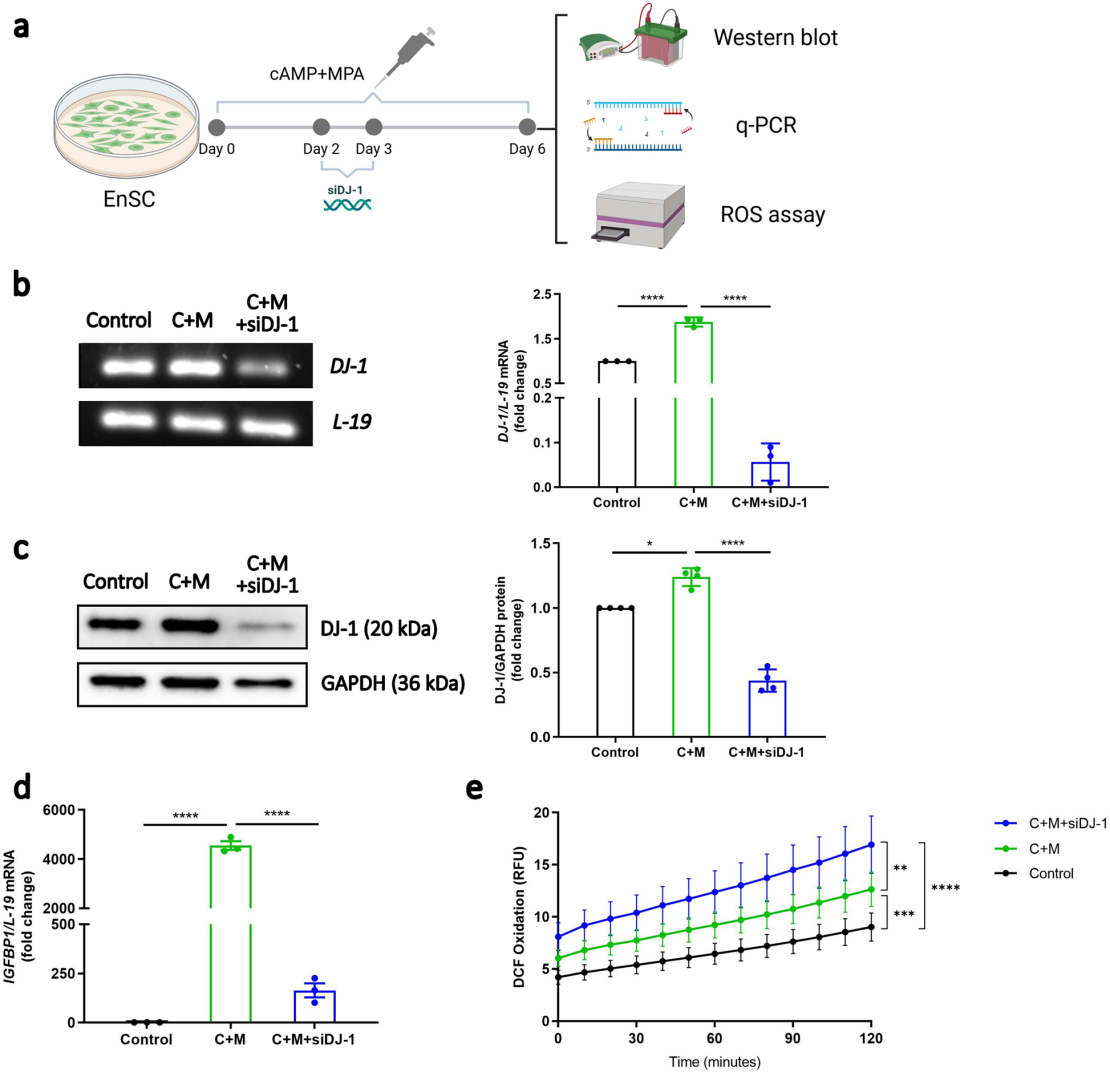
Impact of DJ-1 knockdown on decidualization. **a** Schematic depiction of the experimental approach. **b ***DJ-1* mRNA level and **c** DJ-1 protein level in EnSC transfected by DJ-1-targeting siRNA (siDJ-1) for 24 h, which was performed on the third day of a total of 6-day treatment with 0.5 μM 8-Br-cAMP and 1 μM MPA (C + M) (*n* = 3, *n* = 4). **d ***IGFBP1* mRNA expression level in decidualizing EnSC following transfection of siDJ-1 (*n* = 3). The data are presented as fold change and mean ± SEM. **e** ROS DCFDA fluorescence intensity (raw values) at the indicated time points. The data are presented as mean ± SEM. One-way ANOVA was used to calculate statistical significance. *P < 0.05, **P < 0.01, ***P < 0.001, ****P < 0.0001

Decidualization serves as a crucial preparatory stage for pregnancy establishment. To explore the precise role of DJ-1 in endometrial function we next silenced DJ-1 in EnSC by use of siRNA-mediated gene silencing (Fig. 2a). As depicted in Fig. 2b and c, siRNA-mediated knockdown resulted in a significant reduction of DJ-1 expression at both the transcript (*P* < 0.0001) and protein level (*P* < 0.0001) compared with decidualized EnSC. Notably, in comparison with decidualized EnSC, the deficiency of DJ-1 also elicited a significant decrease in the expression of the decidual marker *IGFBP1* (*P* < 0.0001; Fig. 2d). Furthermore, we also observed a reduction in cell proliferation marker Ki-67 (*P* = 0.0629) and this was further confirmed with a BrdU ELISA (Supplemental Fig. S2a, b; *P* = 0.0299).

DJ-1 acts as a sensor for oxidative stress and it exerts antioxidant effects through multiple mechanisms such as ROS scavenging (Zhang et al. 2020). The loss of DJ-1 contributes to the development of oxidative stress-related diseases (Honbou et al. 2003; Kahle et al. 2009; Eberhard and Lammert 2017; Joselin et al. 2012). We postulated that loss of DJ-1 will lead to elevated ROS levels and impair the induction antioxidants or ROS scavengers. To test this hypothesis, we assessed intracellular ROS production using the H2-DCFDA assay (Gomes et al. 2005). Fig. 2e illustrates that, in comparison with decidualized EnSC, DJ-1 knockdown resulted in a higher level of intracellular ROS production (*P* = 0.0029). We then tested whether the loss of DJ-1 could impair the induction of antioxidants. As a result, *GPX3* and Glutaredoxin (*GLRX*), which functions in the detoxification of hydrogen peroxide, was increased upon decidualization (Lucas et al. 2020; Salker et al. 2011) (*P* < 0.0001; *P* = 0.0386) and was further observed to be downregulated in EnSC when targeted with siDJ-1 (Supplemental Fig. S2c; *P* < 0.0001; *P* = 0.0176) and in keeping, then resulted in cell death (Supplemental Fig. S2d; *P* = 0.013) when compared with the control.

**Fig. 3:**
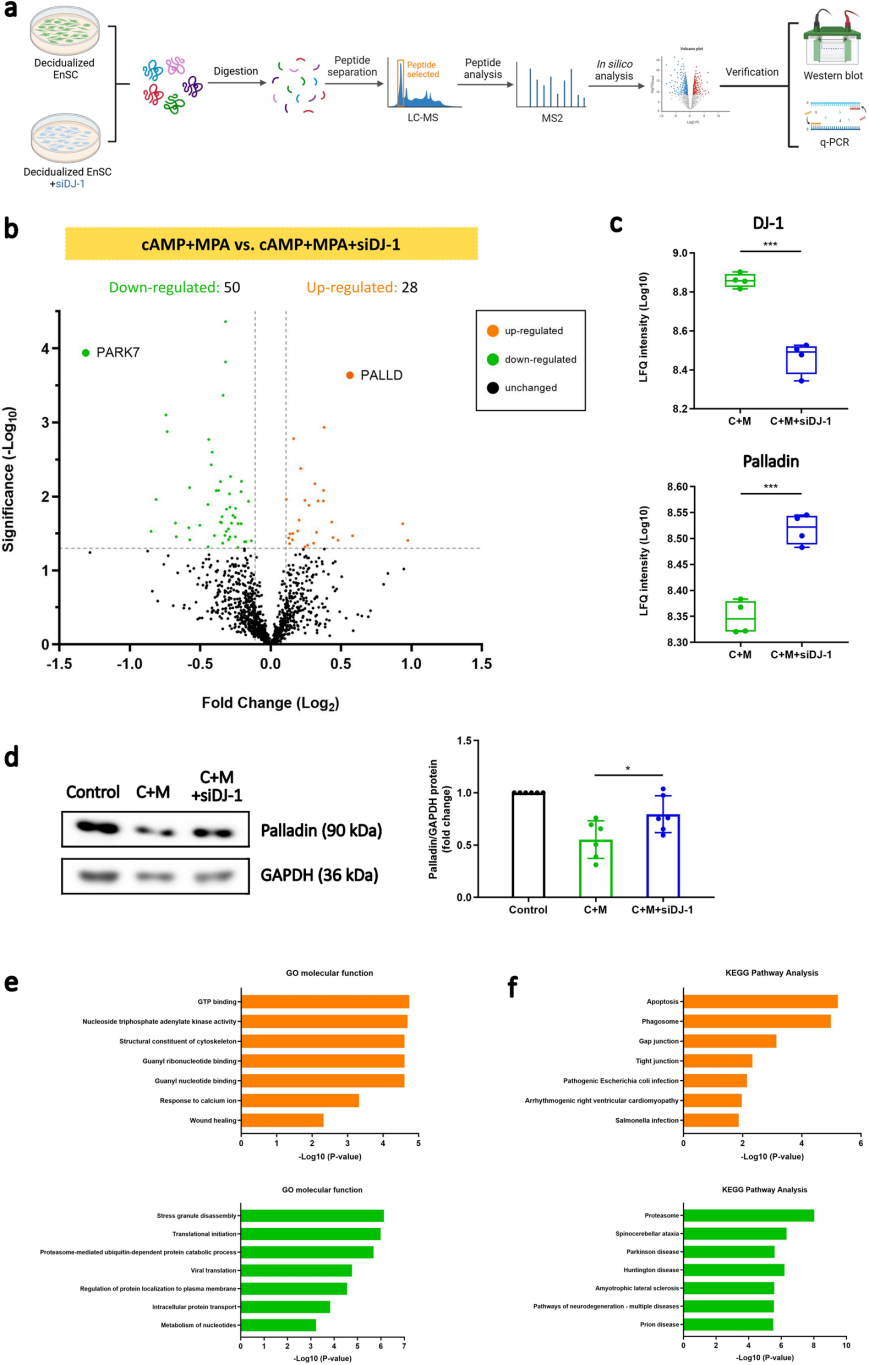
Loss of DJ-1 increases the expression of cytoskeletal protein Palladin. **a** Schematic depiction of the experimental approach. **b** Volcano plots of proteomic data. Results for decidualized EnSC with or without siDJ-1 transfection are shown (*n* = 4). Orange circles show proteins which have significant increases. Green circles show proteins which have significant decreases. Black circles are proteins without any differences. Significantly regulated proteins were defined by a log₂ fold change > 0.11 or < –0.11 and raw p‑value < 0.05. **c** Expression of DJ-1 and PALLD in decidualized EnSC with or without siDJ-1 transfection, presented as LFQ intensity. Box represents the interquartile range (IQR), line indicates the median, whiskers show 1.5 × IQR, unpaired t-test was used to calculate statistical significance. ***P < 0.001. **d** Western blot analysis of Palladin expression in decidualizing EnSC with or without transfection of siDJ-1 (*n* = 6). GAPDH was used as a loading control. The data are presented as mean ± SEM. One-way ANOVA was used to calculate statistical significance. *P < 0.05. **e** GO enrichment analysis of differentially expressed proteins. GO molecular function enrichment analyses of up-regulated (orange) and down-regulated (green) proteins were performed. **f** KEGG enrichment analysis up-regulated (orange) and down-regulated (green) proteins were performed

The known function of endometrial DJ-1 is sparse. To identify novel downstream targets of DJ-1, proteomics (mass spectrometry, LC–MS/MS) was employed to explore differentially expressed cellular proteins in decidual EnSC with or without DJ-1 knockdown (Fig. 3a). As a result, a total of 28 significantly upregulated (orange) and 50 downregulated (green) proteins were observed (Fig. 3b). Upon loss of DJ-1, of the significantly upregulated proteins were actin and cytoskeleton-associated proteins, such as Integrin subunit alpha 5 (ITGA5; *P* = 0.0339), Palladin and matrix metallopeptidase 14 (MMP14; *P* = 0.0389). The significantly downregulated proteins included thioredoxin reductase 1 (TXNRD1; *P* = 0.0662), thioredoxin related transmembrane protein 3 (TMX3; *P* = 0.0013), and GLRX (*P* = 0.0037), which are involved in redox signaling (Supplemental Fig. S3a). As shown in Fig. 3c, the protein level of DJ-1 was significantly decreased upon DJ-1 knockdown, which is consistent with the results of western blotting (*P* = 0.0008). Surprisingly, the level of Palladin, a cytoskeletal protein, demonstrated a significant increase in the DJ-1 knockdown group compared with the control (*P* = 0.0002). Western blotting was conducted in independent experiments verified the upregulation in protein levels of 90 kDa Palladin (P = 0.0282; Fig. 3d and Supplemental Information). Gene Ontology (GO) analysis revealed molecular functions upregulated after DJ-1 knockdown are associated with cell adhesion molecule binding, GTP binding, the structural constituent of the cytoskeleton and NADPH:quinone reductase activity (Fig. 3e). Additionally, Kyoto Encyclopedia of Genes and Genomes (KEGG) analysis indicated the involvement of signaling pathways related to tight junctions and gap junctions after DJ-1 knockdown (Fig. 3f).

Given our finding that Palladin operates downstream of DJ-1, we further investigated whether Palladin also plays a role in early pregnancy. Firstly, we confirmed the presence of PALLD in the endometrium. Single-cell sequencing analysis revealed PALLD gene expression in human endometrial decidua cells (Supplemental Fig. S3b,c) (Vento-Tormo et al. 2018) and Palladin protein expression was likewise observed in both endometrial stromal and glandular cells according to the protein atlas (Supplemental Fig. S3d) (Uhlen et al. 2005). Notably, Palladin expression decreases during decidualization, reaching its lowest level during the window of implantation (Supplemental Fig. S3e). Furthermore, mRNA levels of Palladin were also reduced during early pregnancy in mice (Supplemental Fig. S3f). To elucidate its potential role in pregnancy loss, we conducted western blot to investigate Palladin protein expression in human endometrial tissues. As depicted in Supplemental Fig. S3g and Supplemental Information (original blots), Palladin exhibited higher levels although not reaching significance in endometrial tissue obtained from patients experiencing RPL compared with the control.

### Loss of DJ-1 induces dynamic cytoskeletal change: impact on actin polymerization, cell stiffness, and cell migration

**Fig. 4:**
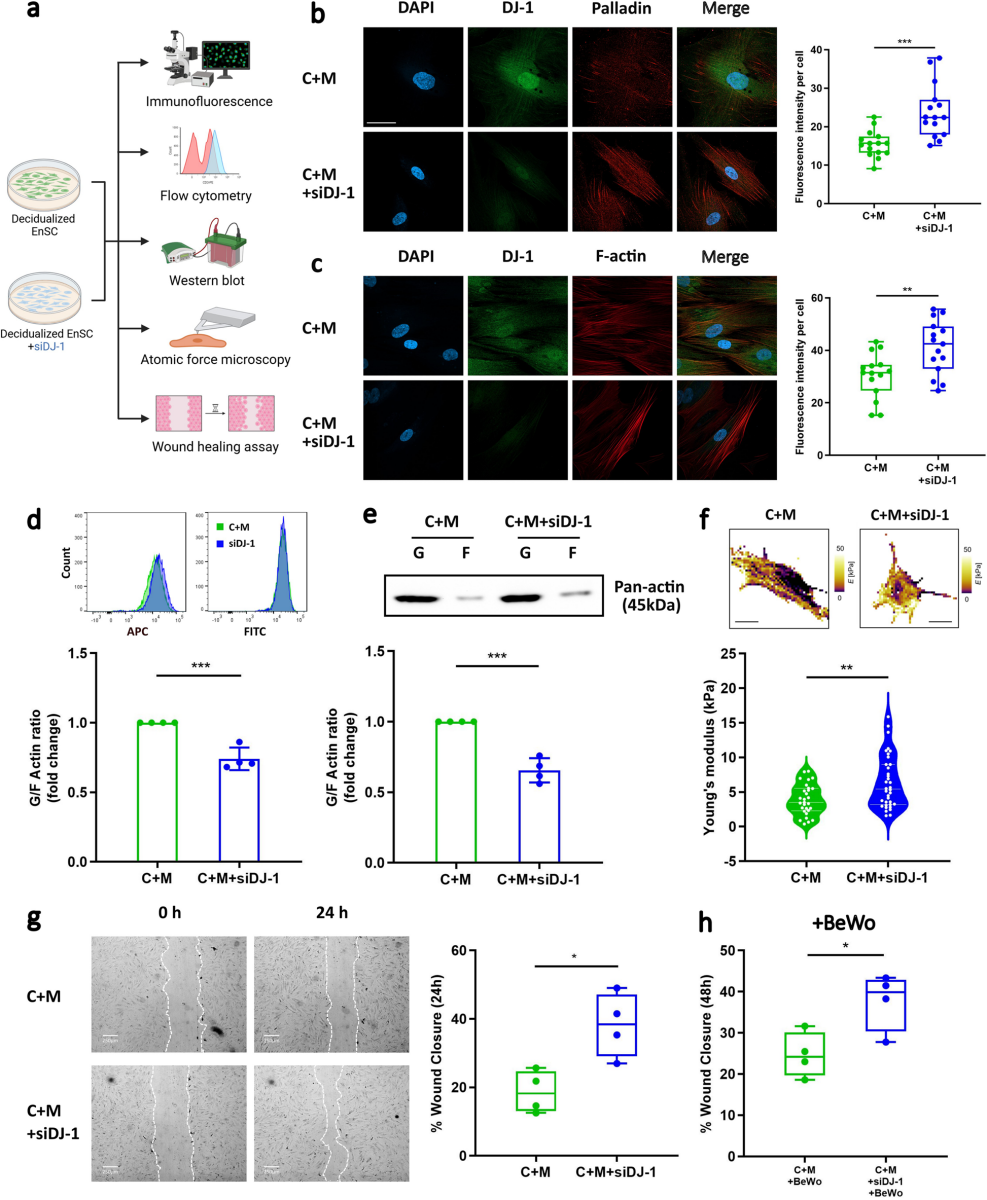
Loss of DJ-1 cause cell cytoskeleton and dynamics change. **a** Schematic depiction of the experimental approach. **b** Immunofluorescence staining of decidualized EnSC with siDJ-1 transfection showed cell morphological changes (upper panel). DJ-1: Alexa Fluor 488 (green); Palladin: Alexa Fluor 568 (red); nucleus: DAPI (blue), (*n* = 3), Scale bar (75 μm). **c** Immunofluorescence staining of decidualized EnSC with siDJ-1 transfection showed cell morphological changes (lower panel). DJ-1: Alexa Fluor 488 (green); nucleus: DAPI (blue); F-actin: Phalloidin (red), (*n* = 3), Scale bar (75 μm). Quantification of immunofluorescence intensity performed from 3 experiments with 15 cells quantified for each condition. Box represents the interquartile range (IQR), line indicates the median, whiskers show 1.5 × IQR. **d** Representative original histogram of Phalloidin (APC, F-actin; Left) and DNase1 (FITC, G-actin; Right) and fluorescence-activated cell sorting analysis (FACS) (*n* = 4), **e** Representative western blot and quantitative densitometric analysis (*n* = 4) of globular/fibrillar actin ratio (G/F-actin ratio) in decidualized EnSC with or without siDJ-1 transfection. The data are presented as mean ± SEM. **f** Image of cell stiffness in decidualized EnSC with or without siDJ-1 transfection measured by AFM and Young’s modulus was measured. Scale bar (20 μm). Violin shows data distribution *via* kernel density, with median and IQR indicated by embedded box plot. **g** Representative images of scratched and recovering wounded areas (marked by white lines) on confluent monolayers of EnSC at different time points (*n* = 4) scale bar (250 μm). Percentage wound closure was determined by measuring the area of the wounds. **h** The percentage of wound closure in scratched decidualized cultures treated with BeWo cell supernatant was determined by measuring the area of the wounds. Box represents the interquartile range (IQR), line indicates the median, whiskers show 1.5 × IQR. The data are presented as mean ± SEM. All results were tested with unpaired t-test to calculate statistical significance. *P < 0.05, **P < 0.01, ***P < 0.001

Palladin functions as an actin-binding and actin-crosslinking protein with effects on cell morphology, mobility and force generation (Azatov et al. 2016; Jin et al. 2009). Here we sought to explore whether DJ-1 also plays a regulatory role in these cytoskeleton dynamics-related processes (Fig. 4a). To assess if there is a putative link between DJ-1 deficiency and the cytoskeleton, immunofluorescence staining was performed on decidualized EnSC, as well as on decidualized EnSC following transfection with siDJ-1.

Globular (G)-actin, the monomeric form of actin, polymerizes into filamentous (F)-actin, serving as a scaffold and cross-linker (Gao and Nakamura 2022). Palladin functions importantly as a cytoskeletal scaffolding protein by functioning as a F-actin-binding protein. As depicted in Fig. 4b and 4c, compared with the rounded morphology observed in decidualized EnSC, the depletion of DJ-1 led to a spindle-like deformation in EnSC, concomitant with increased staining intensity for Palladin (*P* = 0.0002) and F-actin (*P* = 0.0039). Notably, DJ-1 was found to be partially co-localized with Palladin and F-actin. Palladin mainly localized in a punctate pattern along the stress fibers.

Previous research has shown that deregulated Palladin levels causes change in actin reorganization (Dixon et al. 2008). To determine whether the loss of DJ-1 influences the G/F actin ratio, FACS and western blot analyses were employed. As depicted in Fig. 4d, e and Supplemental Fig. S4a and b, the deficiency of DJ-1 led to a significant decrease in the G/F actin ratio, indicating enhanced actin polymerization (*P* = 0.0007; FACS, *P* = 0.0002; WB). Given a previous study indicating a potential relationship between actin polymerization and cell stiffness alteration (Salker et al. 2016), we investigated the association of DJ-1 with cell stiffness using AFM on live EnSC subjected to a 6-day decidualized treatment with or without DJ-1 knockdown. As illustrated in Fig. 4f, the loss of DJ-1 in decidual EnSC resulted in a significant increase in the Young’s modulus (*P* = 0.0026). Taken together these results reveal that DJ-1 loss results in increased actin polymerization and cell stiffness.

Our proteomics analysis further revealed reduced levels of the antioxidant proteins upon DJ-1 knockdown. It has been demonstrated that the generation of ROS can target F-actin and induce changes in the actin cytoskeleton and increase cell motility (Shannon et al. 2022). To test this, we first treated the cells with thymidine to negate the effect of cell proliferation and then performed a cell motility (wound healing) assay. In keeping, we observed that (24-h) post-scratch, the migration of cells was significantly higher in DJ-1 knockdown EnSC compared with the control (*P* = 0.0130; Fig. 4g). Several studies now suggest that the endometrium in women with RPL have increased migration towards aneuploid or abnormal embryos (49, 58). We postulated that part of the ‘defective selection’ process maybe due to loss of DJ-1. In the final set of experiments, we tested if loss of DJ-1 results in more migration if exposed to chemotactic factors derived from aneuploidic cells (BeWo, a common trophoblastic cell line) as a model. As shown in Fig. 4h, the loss of DJ-1 results in a significantly higher aberrant migratory capability when compared with the control (*P* = 0.0255).

### Overexpression of DJ-1 inhibits Palladin expression

**Fig. 5:**
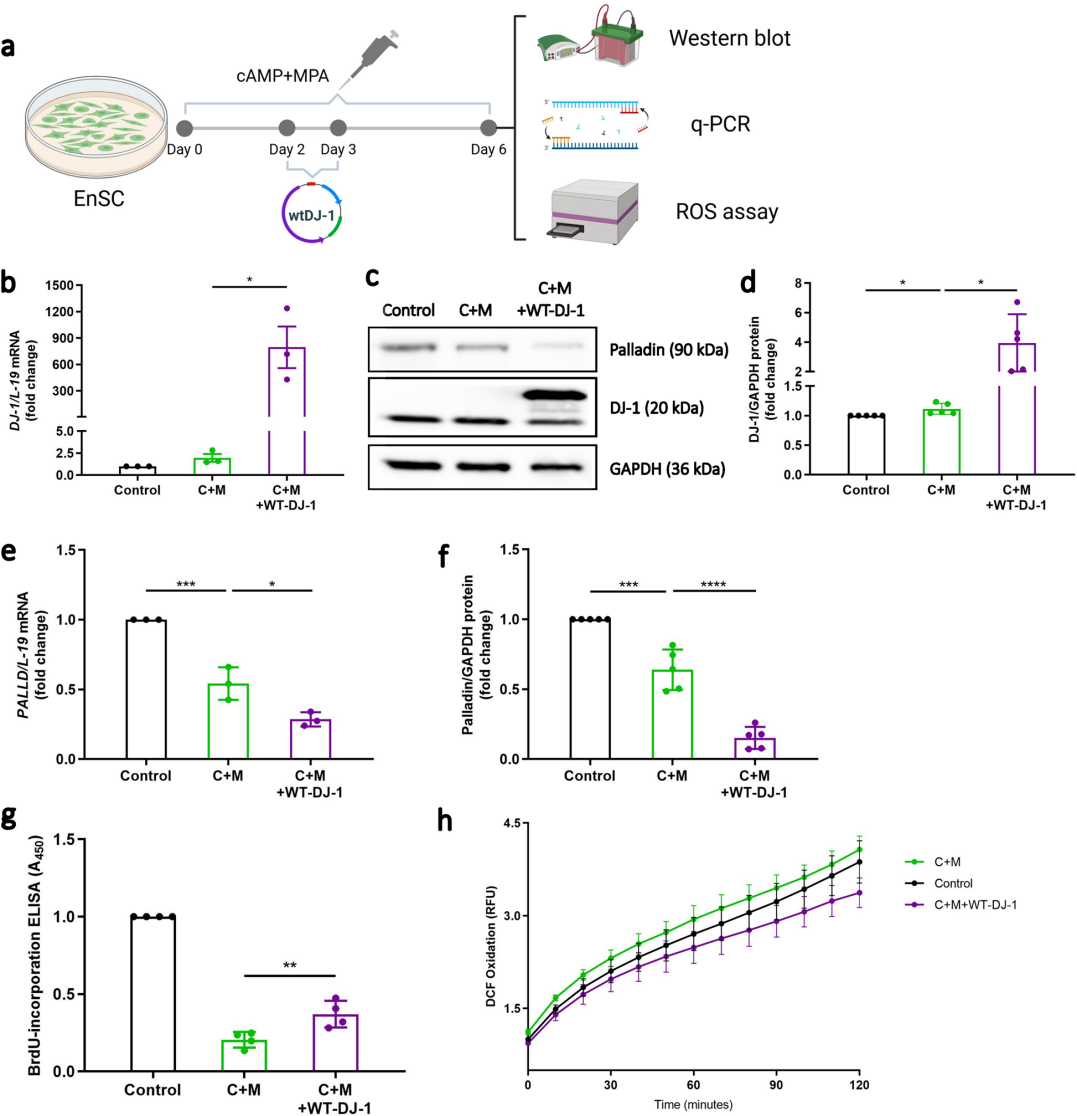
Downregulation of Palladin by DJ-1 overexpression. **a** Schematic depiction of the experimental approach. **b** mRNA expression of *DJ-1* in decidualizing EnSC with transfection of WT-DJ-1 (*n* = 3). **c** Western blot analysis of DJ-1 and Palladin protein in EnSC transfected by pGW1-Myc-DJ-1-wt (WT-DJ-1) for 24 h, which was performed on the third day of a total of 6-day treatment with C + M (*n* = 4). **d** DJ-1 protein level in decidualizing EnSC with transfection of WT-DJ-1. **e ***Palladin* mRNA expression and **f** protein level in decidualizing EnSC with transfection of WT-DJ-1. **g** BrdU ELISA assay was carried out after treatment. Absorbance was measured at 450 nm. **h** ROS DCFDA fluorescence intensity at the indicated time points (*n* = 3). All data are presented as mean ± SEM. One-way ANOVA was used to calculate statistical significance. *P < 0.05, **P < 0.01, ***P < 0.001, ****P < 0.0001

To further investigate whether the effect of DJ-1 overexpression on decidualization could be reversed, EnSC underwent transfection with the pGW1-Myc-DJ-1-wt (WT-DJ-1) plasmid, either in the presence or absence of decidualization treatment (Fig. 5a). As illustrated in Fig. 5b-d, WT-DJ-1 exhibited successful upregulation at both the gene (*P* = 0.0148) and protein (*P* = 0.0305) levels during decidualization treatment. Consequently, the expression of Palladin declined upon decidualization (*P* = 0.0007 q-PCR; *P* = 0.0002, WB), and simultaneous WT-DJ-1 overexpression resulted in a further diminished level of Palladin (*P* = 0.0124, qRT-PCR; *P* < 0.0001, WB; Fig. 5c, e, f). Additionally, the overexpression of DJ-1 during decidualization increased cell proliferation (*P* = 0.0071; Fig. 5g). Furthermore, the H2-DCFDA assay demonstrated that in decidualized EnSC, DJ-1 overexpression led to a decrease in ROS levels (Fig. 5h) below that of control levels although not reaching significance. Further, to evaluate antioxidant expression after WT-DJ-1 we performed qRT-PCR on *GPX3* and *GLRX.* As seen antioxidant levels increase upon transfection (*P* = 0.1759, GPX3; *P* = 0.0069, GLRX; Supplemental Fig. S5).

### Overexpression of DJ-1 inhibits actin polymerization, cell stiffness, and cell migration

**Fig. 6:**
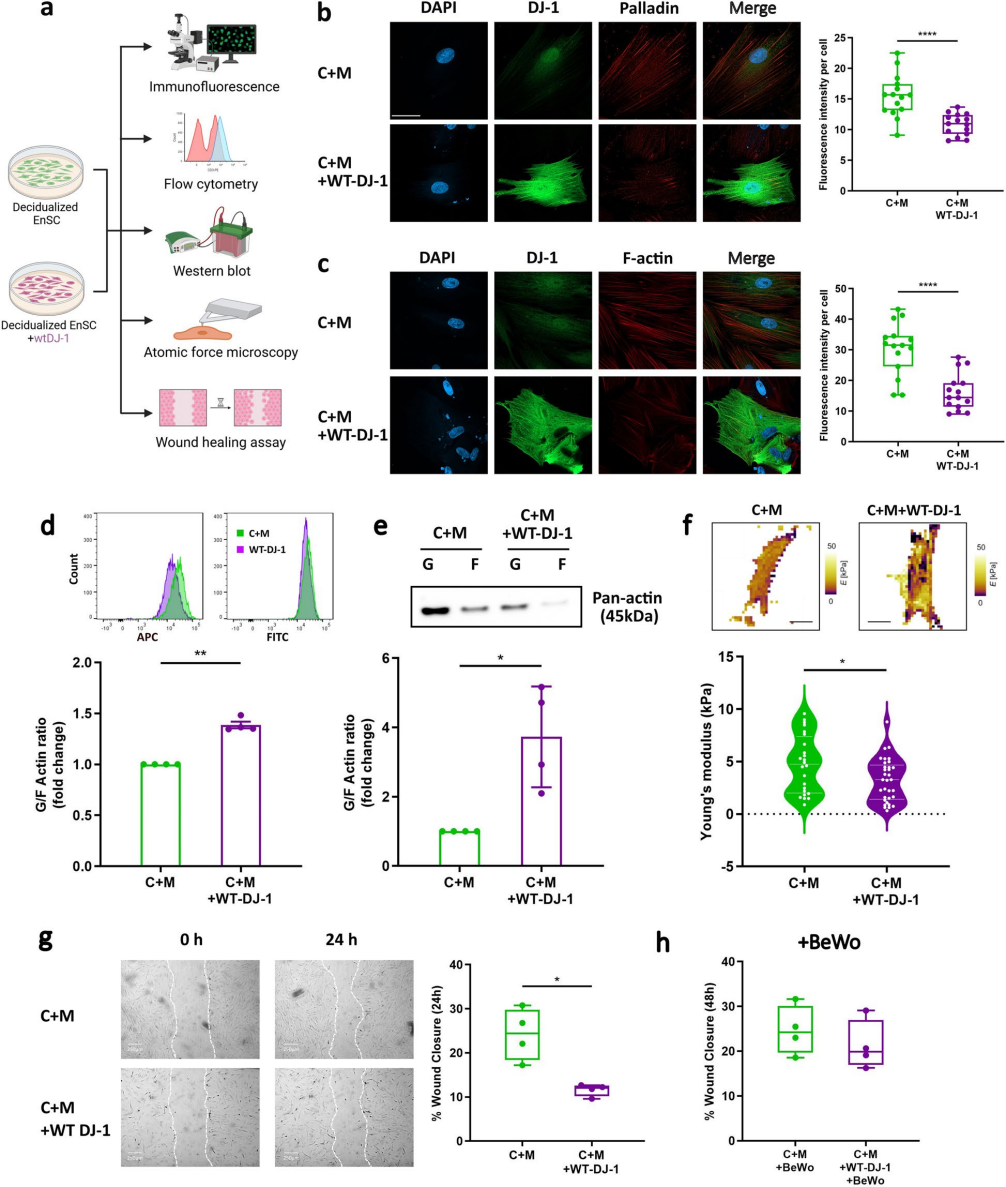
Overexpression of DJ-1 cause cell cytoskeleton and dynamics change. **a** Schematic depiction of the experimental approach. **b** Immunofluorescence staining of decidualized EnSC with WT-DJ-1 transfection showed cell morphological changes (upper panel). DJ-1: Alexa Fluor 488 (green); Palladin: Alexa Fluor 568 (red); nucleus: DAPI (blue); (n = 3). Scale bar (75 μm). **c** Immunofluorescence staining of decidualized EnSC with WT-DJ-1 transfection showed cell morphological changes (lower panel). DJ-1: Alexa Fluor 488 (green); nucleus: DAPI (blue); F-actin: Phalloidin (orange); quantification of immunofluorescence intensity performed from 3 experiments with 15 cells quantified for each condition (*n* = 3). Scale bar (75 μm). Box represents the interquartile range (IQR), line indicates the median, whiskers show 1.5 × IQR. **d** Representative original histogram of Phalloidin (APC, F-actin; Left) and DNase1 (FITC, G-actin; Right) and fluorescence-activated cell sorting analysis (FACS) (*n* = 4), **e** representative Western blot and quantitative densitometric analysis (*n* = 4) of globular/fibrillar actin ratio (G/F-actin ratio) in EnSC with or without WT-DJ-1. The data are presented as mean ± SEM. **f** Images of cell stiffness and Young’s modulus in decidualized EnSC with or without WT-DJ-1. Scale bar (250 μm). Violin shows data distribution *via* kernel density, with median and IQR indicated by embedded box plot. **g** Representative images of scratched and recovering wounded areas (marked by white lines) on confluent monolayers of EnSC at different time points (*n* = 4). Percentage wound closure was determined by measuring the area of the wounds. **h** The percentage of wound closure in scratched decidualized cultures treated with BeWo cell supernatant was determined by measuring the area of the wounds. Box represents the IQR, line indicates the median, whiskers show 1.5 × IQR. Unpaired t-test was used to calculate statistical significance. *P < 0.05, **P < 0.01, ****P < 0.0001

To investigate the association between DJ-1, Palladin and F-actin, immunofluorescence staining was performed with and without WT-DJ-1 transfection (Fig. 6a). As depicted in Fig. 6b and c, overexpression of DJ-1 resulted in reduced Palladin (*P* < 0.0001) and F-actin (*P* < 0.0001) staining intensity. Interestingly, we observed that this reduction in F-actin was particularly pronounced in the perinuclear region and the EnSC lost their characteristic round shape and instead adopted irregular and distorted morphologies.

To further assess actin polymerization, FACS and western blot analyses were employed to determine the G/F actin ratio. As depicted in Fig. 6d, e and Supplemental Fig. S6, the overexpression of DJ-1 led to an increase in the G/F actin ratio, indicating alleviated actin polymerization (*P* = 0.0013, *P* = 0.0331). Additionally, we investigated the association of DJ-1 with cell stiffness using AFM on live EnSC subjected to a 6-day decidualization treatment with or without wt-DJ-1 plasmid transfection. As illustrated in Fig. 6f, the overexpression of DJ-1 in decidual EnSC decreased the Young’s modulus, reflecting a reduction in the cell stiffness (*P* = 0.0266). A wound healing assay was next employed to assess cell mobility. Figure 6g demonstrates that 24h after scratching, the migration of cells to the cell-free region was significantly lower in the context of wt-DJ-1 compared with the control (*P* = 0.0203). Furthermore, migration to abnormal chemotactic signals were not modified upon DJ-1 overexpression (Fig. 6h) indicating that DJ-1 in part controls actin polymerisation and thus endometrial cell migration.

### Validation of DJ-1-Palladin axis as a key mediator of actin dynamics

**Fig. 7:**
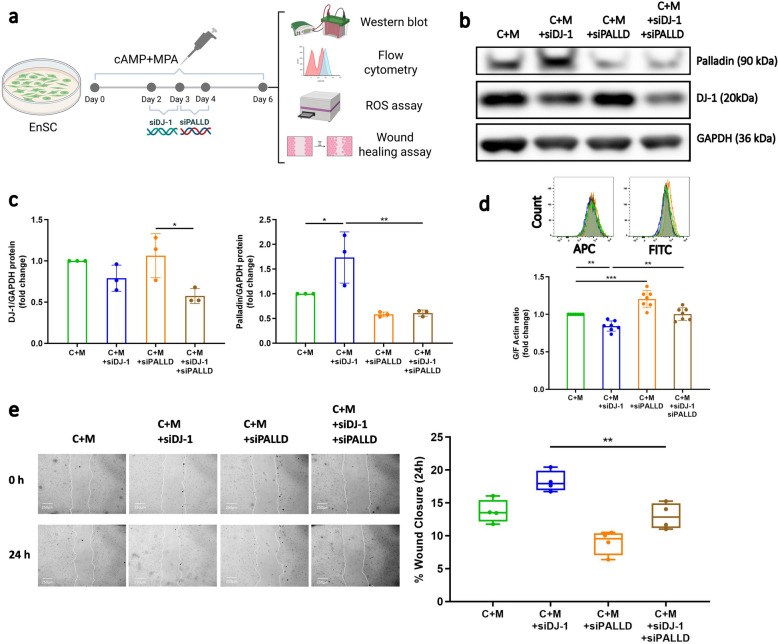
Impact of *PALLD* knockdown on decidualization EnSC following the loss of DJ-1. **a** Schematic depiction of the experimental approach. **b** Western blot analysis of Palladin and DJ-1 expression in decidualized EnSC with or without transfection of siPALLD following DJ-1 knockdown (*n* = 3). GAPDH was used as a loading control. **c** Protein expression of Palladin and DJ-1 in decidualized EnSC with or without transfection of siPALLD following DJ-1 knockdown (*n* = 3). GAPDH was used as a loading control. **d** Representative original histogram of Phalloidin (APC, F-actin; Left) and DNase1 (FITC, G-actin; Right) and flow cytometric analysis of G/F-actin ratio in decidualized EnSC with or without transfection of siPALLD following DJ-1 knockdown (*n* = 7). The data are presented as mean ± SEM. **e** Representative images of scratched and recovering wounded areas (marked by white lines) on confluent monolayers of EnSC at different time points (*n* = 4) scale bar (250 μm). The percentage of wound closure in scratched decidualized cultures treated with BeWo cell supernatant was determined by measuring the area of the wounds. Box represents the interquartile range (IQR), line indicates the median, whiskers show 1.5 × IQR. One-way ANOVA was used to calculate statistical significance. *P < 0.05, **P < 0.01, ***P < 0.001

To verify the role of Palladin as a critical downstream target of DJ-1 controlling F-actin, EnSC were transfected with siRNA *DJ-1*, with or without siRNA-*PALLD* in the presence of decidualization treatment (Fig. 7a). As illustrated in Fig. 7b and c, PALLD siRNA-mediated knockdown (siPALLD) resulted in a 42% reduction of Palladin expression (*P* = 0.2819); in the presence of siDJ-1 transfection (double knockdown) there was a 64% reduction (*P* = 0.0034). As noticed in the above experiments, the DJ-1-Palladin axis plays an important role in actin polymerisation and control of migration. Therefore, we then examined whether loss of Palladin was the key mediator resulting in higher cell polymerization and cell mobility upon loss of DJ-1 by using a double knockdown approach. Using siPALLD, we observed higher levels of G/F actin ratio in the presence of decidualization treatment, which is consistent with Palladin’s role as an F-actin-binding protein (*P* = 0.0004, *orange*). Furthermore, double knockdown (siDJ-1 + siPALLD) led to an increase in the G/F actin ratio (i.e., indicating alleviated actin polymerization, *P* = 0.0047; *brown*), supporting the notion that Palladin is indeed the key player in controlling polymerization (Fig. 7d). Additionally, to confirm whether changes in F-actin could modify migration, we performed a wound healing assay in the presence of chemotactic factors derived from aneuploidic trophoblast cells (conditioned media). As seen in Fig. 7e, the loss of Palladin in combination with DJ-1 knockdown led to a significant reduction of cell mobility, with levels being restored to that of C + M (*P* = 0.0066), illustrating that DJ-1-Palladin axis can modulate migration to aneuplodic signals. However, knockdown of *PALLD* alone reduced the level of intracellular ROS production compared with siDJ-1 (*P* = 0.021); double knockdown did not have a discernible effect (Supplemental Fig. S7).

**Fig. 8:**
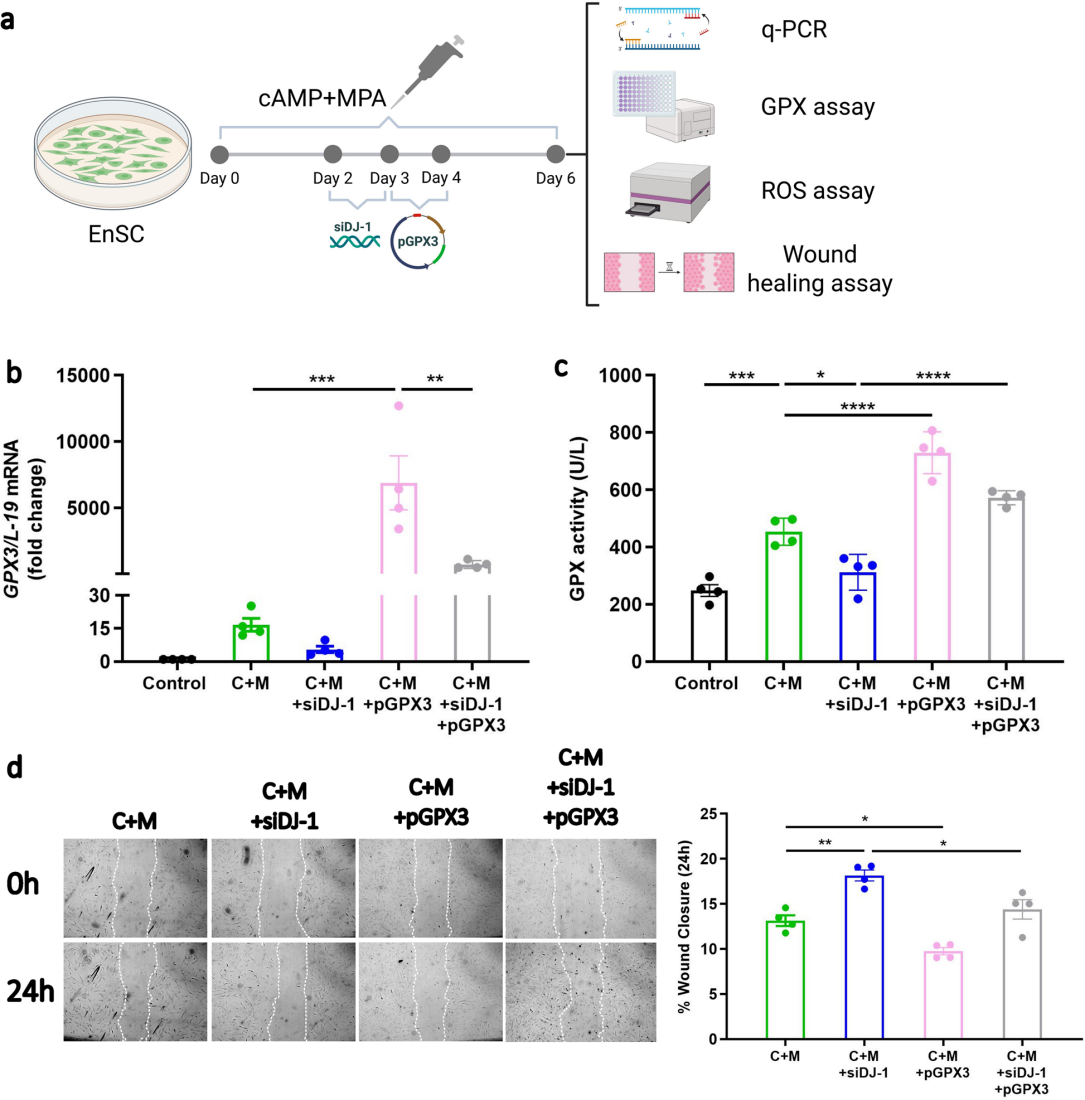
GPX3 overexpression rescues the aberrant effect caused by the loss of DJ-1. **a** Schematic depiction of the experimental approach. **b*** GPX3* mRNA level in decidualized EnSC with or without transfection of pGPX3 following DJ-1 knockdown (*n* = 4) confirming transfection efficiency. Ct values were normalized to the housekeeping gene *L-19*. **c** Glutathione peroxidase (GPX) activity in decidualized EnSC with or without transfection of pGPX3 following DJ-1 knockdown (*n* = 4). **d** Representative images of scratched and recovering wounded areas (marked by white lines) on confluent monolayers of EnSC at different time points (*n* = 4) scale bar (250 μm). The percentage of wound closure in scratched decidualized cultures treated with BeWo cell supernatant was determined by measuring the area of the wounds. The data are presented as mean ± SEM. One-way ANOVA was used to calculate statistical significance. *P < 0.05, **P < 0.01, ***P < 0.001, ****P < 0.0001

Actin polymerization and bundling are processes that can be affected by ROS (Shannon et al. 2022). The reduction of hydrogen peroxide is catalysed by GPX3 through the conversion of GSH to GSSG (Forman et al. 2009; Prabhakar et al. 2005). Glutathione reductase (GR) then regenerates GSH from GSSG *via* consumption of NADPH, thus preventing oxidative damage and sustaining the redox equilibrium (Forman et al. 2009; Pei et al. 2023). As shown above, loss of DJ-1 reduces GPX3 levels thus concomitantly increasing ROS levels. In the final set of experiments, to elucidate the link between DJ-1 and ROS, we investigated the interplay of GPX3 antioxidant behaviour. Therefore, GPX3 plasmid (pGPX3) was transfected to decidualized EnSC with or without siDJ-1 for 24h to induce GPX3 overexpression (Fig. 8a). As a result, we observe that *GPX3* mRNA levels are significantly induced both in the absence (*P* = 0.0007) and presence of siDJ-1 (*P* = 0.9789, Fig. 8b). In parallel, the supernatant from decidualized EnSC with siDJ-1 or pGPX3 was collected and GPX3 activity was measured. As seen in Fig. 8c, loss of DJ-1 resulted in lower GPX3 activity (*P* = 0.0128), overexpression of GPX3 (alone) resulted in higher activity (*P* < 0.0001) as expected, and GPX3 overexpression together with DJ-1 knockdown rescued GPX activity (*P* < 0.0001). Since GPX3 is an antioxidant, we further measured the ROS level and cell motility. As shown in Supplemental Fig. S8, overexpression of GPX3 mitigated the abnormal high ROS level caused by the loss of DJ-1 (*P* = 0.0161) by 60 min and in parallel reduced the migration capacity in the presence of chemotactic factors derived from aneuploidic trophoblast cells (*P* = 0.0132; Fig. 8d).

## Discussion

Human decidualisation is unique as it occurs spontaneously 6 days after ovulation independent from an implanting embryo and heralds the closing of the implantation window (Gellersen et al. 2007; Ochoa-Bernal and Fazleabas 2020). This intricate process is orchestrated by postovulatory progesterone and cAMP signaling (Lavogina et al. 2021). In EnSC, decidualization is triggered by an acute stress response, causing a burst of ROS and secretion of proinflammatory cytokines and silencing of cell cycle genes and stress pathways (Okada et al. 2018). Subsequently, EnSC are highly resistant to stress signals (i.e. oxidative and metabolic stressors) and remain impervious to harmful (micro-)environmental signals (Okada et al. 2018). The decidua then forms a semblant-matrix and tolerogenic environment encapsulating the implanting embryo (Ochoa-Bernal et al. 2020). Approaching the 12th week of gestation, the trophoblast plugs in the spiral arteries are removed which coincides with a significant rise in oxygen levels (from ~ 20 mmHg to ~ 40 mmHg) (Brosens et al. 2022). The resulting surge in ROS serves as a ‘stress test’ for the integrity of the placental-decidual interface (Zhang et al. 2023). Therefore, the induction of a robust antioxidant defence is pivotal to a successful pregnancy.

Human DJ-1 is a widely expressed antioxidant protein that plays a vital role in various physiological functions (Kahle et al. 2009) and has been extensively characterised in Parkinson’s disease. Our study confirmed the presence of DJ-1 in the endometrium and revealed its dynamic expression pattern during decidualization, observed in both EnSC and in a murine gene-knockout model. ROS are by-products of aerobic respiration and metabolism and are essential for key biological processes such as gene expression, proliferation, cell differentiation and motility (Finkel 2011). Moderate levels of ROS induce cellular adaptations to stress or resilience, a conserved (both in pro- & eukaryotes) response known as ‘*hormesis*’ (Nitti et al. 2022). Hormesis describes a biological adaptive response in which mild stress exposure to cells induces protection against subsequent more intense insults (Nitti et al. 2022). Dröge reported that an oxidative burst is critical to ‘kick-start’ the redox defence mechanism and inducing the expression of genes encoding for antioxidants (Droge 2002). In approximately 70% of first-trimester losses, the cytotrophoblastic ‘crown’ surrounding the conceptus is thin and fragmented and there is strong evidence of oxidative injury (Brosens et al. 2022). In keeping, we observed that DJ-1 creates a protective ‘crown’ around the conceptus. Further, in the female *Dj-1*^*−/−*^ implantation sites we observed that the decidual cells were disorganized, the villi formation was delayed, and the villi were thicker with fewer blood vessels in Dj-1 knockout female mice during early pregnancy. We also provide evidence that a key antioxidant enzyme, GPX3 is lost and could make the embryo vulnerable to oxidative insults. Both euploid and aneuploid losses share these pathological features, indicating they may result from either an abnormal decidual microenvironment or from impaired protection from ROS created from byproducts of aerobic respiration and metabolism (Brosens et al. 2022).

Oxidative stress (OS), characterized by elevated levels of ROS (superoxide (O2^−^), hydrogen peroxide (H_2_O_2_) and hydroxyl radicals (OH^−^), is one of the key factors influencing decidualization (Agarwal et al. 2008; Ruder et al. 2008). cAMP may enhance endogenous ROS production, which occurs alongside a rise in IGFBP1 expression during decidualization (Sugawara et al. 2000; Al-Sabbagh et al. 2011). However excessive ROS disrupts decidualization, triggering stromal cell apoptosis and thus leading to a decrease in implantation sites (Hussain et al. 2021; Shahin et al. 2013). The increasing levels of DJ-1 correspond to rising levels of ROS, functioning to regulate ROS levels within physiological ranges conducive to normal decidualization. Conversely, loss of DJ-1 elevates ROS levels, consequently disrupting the cAMP/ROS/IGFBP1 signaling pathway and impairing decidualization. We show that DJ-1 is sensitive to cAMP signaling and the loss of DJ-1 results in significantly lower levels of IGFBP1 and cell death in keeping with the hypothesis. In normoxic conditions, DJ-1 is predominantly found in the cytoplasm; however, upon exposure to OS, it can translocate from the cytoplasm to the mitochondria and subsequently to the nucleus (Junn et al. 2009). The distinct subcellular localization of DJ-1 aligns with findings that intracellular ROS generation is spatially restricted, typically confined to the specific subcellular sites where free radicals mediate physiological signaling (Kaludercic et al. 2014). The mitochondrial-localized DJ-1 is important in short-term protection against OS, after which it translocates to the nucleus (Zhou et al. 2020). Our findings further support this dynamic behaviour, as we observed heightened nuclear localization of DJ-1 following decidualization.

Several studies suggest that the endometrium of those experiencing RPL displays a prolonged and dysregulated pro-inflammatory decidual reaction (Salker et al. 2012). This exaggerated inflammatory response prolongs the window of receptivity, leading to implantation beyond the window of implantation and disrupting embryo selection (Salker et al. 2010). Furthermore, the absence of implantation ‘quality control’ presents as a rapid time-to-conception, known as 'superfertility' phenomenon often observed in many RPL patients (Bhandari et al. 2016; Teklenburg et al. 2010). In our investigation, utilizing midluteal endometrial RNA-sequencing data from controls and those with RPL, we observed a significant reduction in *DJ-1* gene expression in individuals with RPL. Given that the imbalance of ROS can lead to inflammation (Hussain et al. 2016), these results indicate a possible antioxidant role for DJ-1 in the pathogenesis of RPL. This finding was further supported by western blot analysis, which demonstrated a corresponding reduction in DJ-1 protein expression in the endometrium of women suffering from RPL. Future studies are required in larger cohorts to verify if DJ-1 can be a useful biomarker. Furthermore, in mice, within the implantation window, we observed the localization of DJ-1 surrounding the implantation site (crown like), suggesting a potential involvement of DJ-1 in the endometrium supporting decidualisation by creating a semi-independent, anti-inflammatory network surrounding the embryo during early gestation (Diniz-da-Costa et al. 2021). As described in a previous study, reduced expression of Foxp3 and regulatory T cells (Tregs) induction in DJ-1 knock out mice, this may also further exacerbate and immune homeostasis during pregnancy resulting in pregnancy loss (Singh et al. 2015). Notwithstanding, our in vivo murine model, we observed a notable reduction in litter size and abnormal placentation among female mice lacking DJ-1, indicating a potential role of maternal DJ-1 in maintaining a normal pregnancy.

As a previous study reported, DJ-1 is essential in controlling metastasis in endometrial cancer and endometriosis, which is associated with actin-regulating signaling pathways (Jin et al. 2020). In our present study, using proteomics, we found that the actin-associated protein Palladin is upregulated upon DJ-1 knockdown during decidualization. Palladin is a member of a small group of immunoglobulin (Ig) domain-bearing proteins, that are found at the Z-line and linked to the F-actin cytoskeleton (Otey et al. 2009). Palladin exists in three main isoforms: the 200-kDa isoform primarily expressed in adult myocardium, skeletal muscle and testes; the 140-kDa isoform largely found in cardiac and smooth muscle; and the 90-kDa isoform, the most common, which is widely expressed across many cell types (Wang and Moser 2008). In a rat model, the 140-kDa isoform was increased significantly in luminal epithelial cells at the time of implantation (Nicholson et al. 2018). In our study, we observe specifically the expression of 90-kDa isoform, which decreases during decidualization. Palladin participates in a variety of actin-related structures—including focal adhesions, stress fibers, cell–cell junctions, and Z-lines which are vital for the formation, arrangement and preservation of the actin cytoskeleton (Otey et al. 2009; Jin 2011). Previous research has observed that *Palladin*^*−/−*^ mouse embryo fibroblasts have elevated G-actin and a reduced F-actin level (Liu et al. 2007). In our findings, we observed that knockdown of Palladin could also elevate G- actin and a reduced F- actin levels. Furthermore, we evaluated Palladin at protein level using human endometrial samples and observed an increase in Palladin levels in the RPL group, although it did not reach significance. We acknowledge that our study included small cohorts, and further studies are needed to validate this finding in larger cohorts. Nonetheless, in keeping with our study, we demonstrated that loss of Palladin can decrease actin polymerization and inhibit cell migration.

In our study, we have shown that DJ-1 has an unexpected but crucial role in maintaining the balance of Palladin expression and regulating cytoskeletal dynamics. These processes are essential for decidualization and migration in early pregnancy. During decidualization, EnSC undergoes morphological and cytoskeleton changes (Pan-Castillo et al. 2018), which are important for the receptive phase of the endometrium and early gestational development. We used immunofluorescence to show that aberrant DJ-1 levels cause deformation in decidual EnSC and loss of DJ-1 can result in the typical round globular decidual shape to adopt an epithelial-like shape, while overexpression of DJ-1 grossly changes the morphology. Consistent with the above results, DJ-1 deficiency causes higher intensity of Palladin, which colocalizes with DJ-1 on stress fibers. Additionally, the higher intensity of F-actin upon DJ-1 knockdown indicates a potential enhancement of actin polymerization. Furthermore, prolonged stabilization of F-actin can have a negative effect on the differentiation of EnSC into decidual cells (Ihnatovych et al. 2009). Our findings show that DJ-1 deficiency during decidualization enhances ROS, actin polymerization and cell stiffness, whereas DJ-1 overexpression has the opposite effect. Additionally, the aberrant actin polymerization induced by DJ-1 deficiency can be rescued by double knockdown of the downstream cytoskeletal regulator Palladin. Therefore, the aberrant actin dynamics and cell stiffness caused by DJ-1 alteration may impair decidualization.

ROS is known for its ability to induce cell mobility and loss of DJ-1 is known to increase ROS levels (Shannon et al. 2022). GPX3 is a known ROS scavenger and is essential for cell homeostasis (Nirgude and Choudhary 2021). Deficiency of GPX3 has been associated with increased expression of epithelial-mesenchymal transition (EMT) markers like Vimentin, alongside reduced expression of the epithelial marker E-Cadherin (Cai et al. 2019). Additionally, GPX3 blocks metastasis through downregulation of MMPs, mediated by suppression of the FAK/AKT signaling pathway (Zhu et al. 2018). Consistent with these findings, our study demonstrates that loss of DJ-1 leads to a decrease in GPX3 expression and an increase in MMP14 levels, leading to aberrant accumulation of ROS and increased migration. This oxidative imbalance is associated with increased cell motility, as observed in our experiments. Moreover, overexpression of GPX3 partially restored ROS levels and attenuated the enhanced cell motility induced by DJ-1 deficiency. However, these effects were modest, suggesting that the functional activity of GPX3 could depend on factors regulated by DJ-1. Additionally, DJ-1 knockdown may activate parallel stress pathways that contribute to oxidative stress or nitric oxides and increase cell motility, which are not fully addressed by GPX3 overexpression alone.

Interestingly, the abnormal migration of EnSC (from RPL women) towards poor-quality embryos, points to an abnormally hyper-receptive endometrial state in women affected by RPL (Weimar et al. 2012). This was further reinforced with our own experimental data, suggesting that loss of DJ-1 resulted in more migration to aneuploidic chemotactic signals (Lucas et al. 2016). The mechanism by which decidualization reprograms EnSC to respond to embryonic fitness signals is not fully understood. Recent data has shown that high-quality blastocysts secrete hsa-miR-320a (MIR320A, a conserved microRNA crucial for pre-implantation development) increases migration (Berkhout et al. 2020). Using miRDB (an online database for miRNA target prediction) reveals a tentative binding site of miR-320 on the 3’UTR on Palladin as well as other actin-related genes. In addition, miR-320 has a known role on actin dynamics (Nguyen and Lee 2022). Therefore, taken together, this increased cell mobility through the reduction of miR-320a may additionally drive the endometrium into a hyper-receptive state, making it more susceptible to implantation of low-quality embryos and ultimately contributing to RPL. Nonetheless, additional research is necessary to confirm this hypothesis.

It has been reported that endometrial cells initially trigger an acute stress response before bifurcating as robust endometrial decidual or primary (acute) senescent cells which are eliminated by uterine natural killer (uNK) cells (Brighton et al. 2017). In a senescent state, cells cease to divide and secrete a range of bioactive molecules, including ROS, ECM proteins and pro-inflammatory mediators and accumulation of β-galactosidase, known as the senescence-associated secretory phenotype (SASP) (Kumari and Jat 2021). Interestingly, loss of DJ-1 allows senescent cell survival following stress (Ozcan et al. 2016), which may be partly due to an increase of ROS and an increase of ECM proteins by promoting the ‘bystander effect’. Our proteomics data also shows an increase in β-galactosidase upon knockdown of DJ-1 (Tables 3 and 4; Pride database: PXD051694). Importantly, a pro-senescent decidual response has been associated with RPL (Lucas et al. 2020). It is plausible to propose that loss of DJ-1 can also result in pro-senescence denoted by a cellular stress response induced by replicative exhaustion or other stressors that lead to macromolecular injury resulting in pregnancy loss (Herr et al. 2024) and future investigations are required to elucidate the role of other cell types (i.e. uNK) in this process.

**Table 3:** Upregulated genes in decidualized EnSC with or without DJ-1 knockdown

Upregulated Gene names	-Log Student's t-test *p*-value non target KD_DJ-KD	Student's t-test Test statistic non target KD_DJ-KD	Student's t-test Difference/LOG2 FC
*SERPINE2*	1,405030577	−2,624425777	−0,974631786
*MARCKSL1*	1,632736062	−3,023394979	−0,939121246
*ITGA5*	1,469650905	−2,736075538	−0,582092762
*PALLD*	3,638092062	−7,824326679	−0,563687801
*MMP14*	1,409666173	−2,632398167	−0,479485989
*EHD1*	1,44477504	−2,847929735	−0,44367822
*ERLIN2*	1,654027191	−3,061560981	−0,434356689
*AK3*	2,932858195	−5,784870736	−0,379790783
*ARMT1*	2,080983827	−3,866569427	−0,376213074
*SLC25A24*	1,940897753	−3,593337836	−0,374184132
*LMNA*	1,939639891	−3,590927842	−0,337780476
*PRMT1*	1,514045084	−2,81345956	−0,325448036
*S100A11*	2,170776171	−4,047032195	−0,315128326
*CTSL*	1,367577724	−2,560210231	−0,305739403
*S100A16*	1,878897827	−3,475422634	−0,272423267
*TP53I3*	1,339608953	−2,51247375	−0,263301849
*UBE2V1;TMEM189-UBE2V1*	1,318409905	−2,476408429	−0,244933128
*CSTB*	1,948312559	−3,60755938	−0,244910717
*LRPPRC*	2,377412481	−4,479729994	−0,213801384
*GLB1*	1,679793428	−3,107968971	−0,202206612
*ATP5O*	1,535363029	−2,850829666	−0,192224979
*SRI*	2,78028695	−5,403440138	−0,163616657
*PGM3*	1,498082541	−2,785568084	−0,156730175
*NONO*	1,414808902	−2,641249196	−0,155093193
*EDF1*	1,497027524	−2,783727329	−0,136477947
*PRDX1*	1,363779573	−2,553717003	−0,135764599
*REEP5*	1,439470863	−2,683790715	−0,128194332
*TUBA1B;TUBA1C;TUBA1A*	1,961926547	−3,633738892	−0,11085701

Upregulated proteins (with -Log10 *p*-value > 1.3) in decidualized EnSC with or without DJ-1 knockdown, ranked by Student's t-test difference

**Table 4:** Downregulated genes in decidualized EnSC with or without DJ-1 knockdown

**Downregulated Gene names**	-Log Student's t-test *p*-value non target KD_DJ-KD	**Student's t-test Test statistic non target KD_DJ-KD**	**Student's t-test Difference/LOG2 FC**
*PSMC4*	1,39974303	2,615338824	0,13684845
*CLTC*	1,933245613	3,578688265	0,15719986
*PSMA7*	1,389744463	2,598174931	0,175521374
*ETF1*	1,387494955	2,594316771	0,183530807
*PSMA1*	2,204200387	4,115335179	0,206454754
*PGRMC1*	2,064071765	3,833058955	0,209489822
*EIF3F*	1,631608402	3,021378068	0,209855556
*RUVBL2*	1,311205642	2,464173979	0,231031418
*IMPDH2*	1,632125205	3,022302356	0,231866837
*ASPH*	1,83969325	3,401756624	0,247897148
*SSB*	1,456198672	2,712739222	0,250386715
*RBBP4;RBBP7*	1,715421974	3,172549041	0,253197193
*EIF3A*	1,742347223	3,221678182	0,267253876
*PFDN2*	1,638894545	3,034417884	0,276625633
*SNX1*	2,268062317	4,247608636	0,284954548
*LRRC59*	1,856256927	3,432797341	0,288261414
*SRP9;DKFZp564M2223*	1,453693262	2,708398548	0,289227486
*CSE1L*	2,035454236	3,77668996	0,290929317
*SCAMP3*	1,560868716	2,895728193	0,298167229
*ACBD3*	1,41803388	2,646803124	0,298781872
*ABCE1*	1,828869561	3,381537222	0,313004971
*PSMB6*	1,529635513	2,840775588	0,314351559
*TMEM43*	3,817922342	8,429175615	0,319987297
*RPS25*	4,362612889	10,51828013	0,320022106
*PSMA4*	1,473491546	2,742747502	0,324090004
*VCP*	3,365279878	6,976610677	0,337694645
*KIF5B*	1,367411614	2,559926182	0,340960979
*MARS*	1,728573469	3,196510423	0,344823837
*KPNA4*	1,64496608	3,605441429	0,34834671
*ATP2A2*	2,200577992	4,10790242	0,355434418
*EIF4H*	1,65444008	3,062302727	0,356203079
*TFG*	2,082162671	3,8689108	0,368225574
*CCBL2*	2,07864586	3,861927996	0,386444569
*PDAP1*	1,472437799	2,740916512	0,398446083
*PSMB1*	2,597936526	4,971187995	0,414880753
*GLRX*	2,427713358	4,589000059	0,42138052
*SYNCRIP*	2,770990184	5,38079684	0,439610481
*KLC1*	1,318148056	2,475963558	0,442950726
*USP14*	1,891362359	3,498987094	0,445399761
*CLINT1*	1,609622221	2,982143023	0,502355099
*SPTBN1*	1,414943049	2,641480165	0,573438644
*GARS*	2,120596624	3,945648026	0,575049877
*SPTAN1*	1,577522916	2,925158442	0,581540585
*IDI1*	1,454317021	2,709479061	0,669998646
*STAT1*	1,641216207	3,038576865	0,674937725
*TMX3*	2,874812424	5,637535352	0,734701157
*TXNRD1*	3,101347242	6,22879191	0,742181778
*AKAP12*	1,957386903	3,624999395	0,812675953
*PYGL*	1,527333378	2,836737298	0,84880209
*PARK7*	3,940141211	8,862895485	1,312864304

Downregulated proteins (with -Log10 *p*-value > 1.3) in decidualized EnSC with or without DJ-1 knockdown, ranked by Student's t-test difference

## Conclusion

**Fig. 9:**
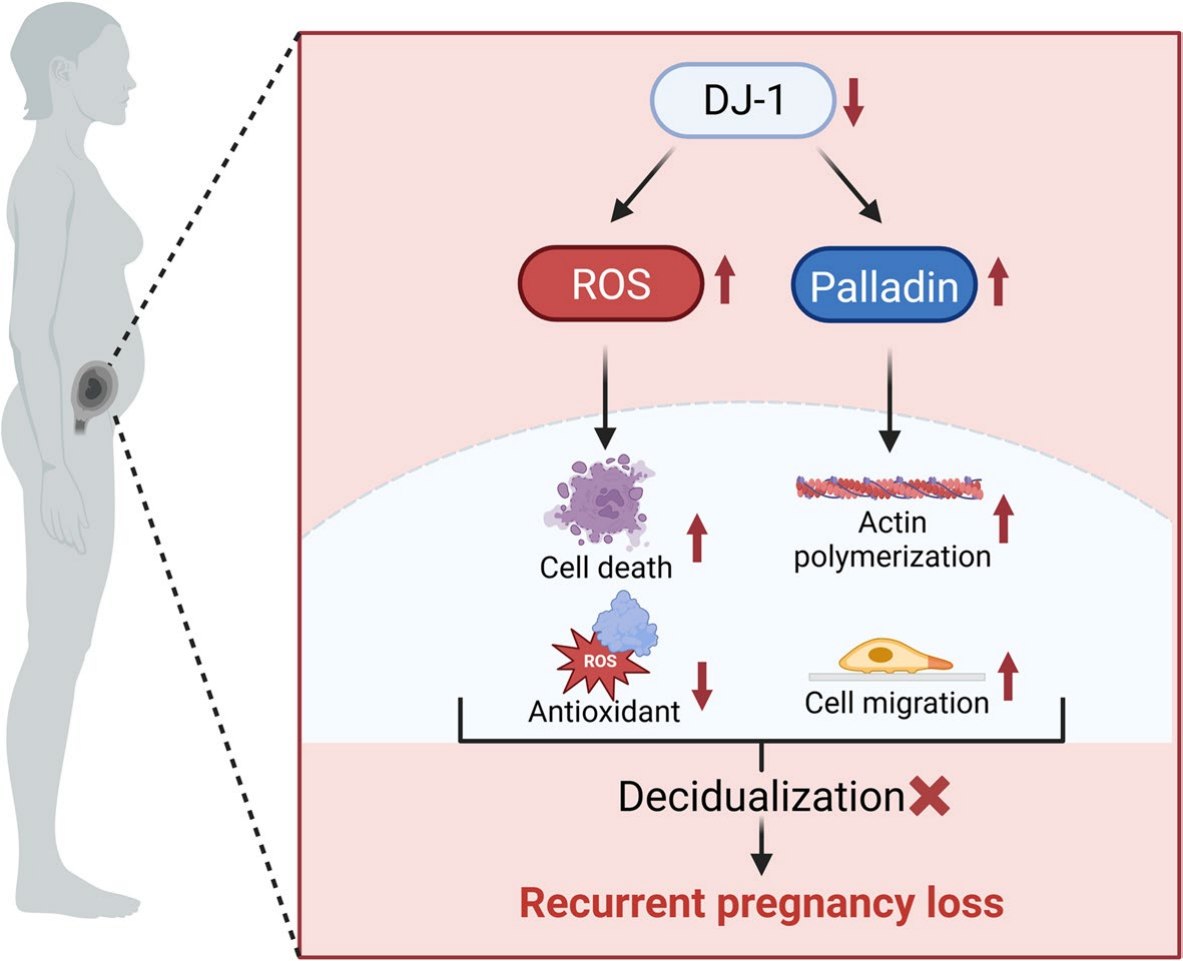
Graphical abstract depicting the intricate role of DJ-1 in endometrial physiology. The absence of DJ-1 resulted in an increase in ROS levels and a decrease in the level of antioxidant enzymes, which further cause an increasing cell mobility and cell death. Additionally, DJ-1 dysregulation caused significant changes in cytoskeletal dynamics, affecting actin polymerization, cell morphology, and migration through Palladin. This led to an impaired decidualization, and is associated with pregnancy loss

In conclusion, decidualization is associated with a burst of ROS followed by induction of antioxidant protective mechanism known as ‘*hormesis*’. By the 12th week of gestation, the trophoblastic plugs are removed, and oxygen tension increases rapidly. The resulting surge in ROS challenges the functional integrity of the placental-decidual interface. We uncovered an unexpected role for DJ-1 in the endometrium during early pregnancy and deficiency of DJ-1 is associated with both murine and human pregnancy loss. The loss of DJ-1 reduced IGFBP1 and increased ROS and cell death. Additionally, dysregulation of DJ-1 induced dynamic changes in cytoskeletal dynamics, influencing actin polymerization, cell morphology and cell migration *via* Palladin. Furthermore, this resulted in an increase of migration to chemotactic signals from aneuploidic trophoblast cells. These effects were reversed by DJ-1 overexpression and partially ameliorated by rebalancing the downstream effectors Palladin and GPX3. In summary, these findings underscore pivotal role of DJ-1 in endometrial ROS handling, cytoskeletal rearrangements, and cell migration during early pregnancy and reveal that deregulation of the DJ-1-Palldin axis is correlated with aberrant migration and recurrent pregnancy loss (Fig. 9).

## Supplementary Information

Supplementary Material 1.

Supplementary Material 2.

Supplementary Material 3.

## Data Availability

Data is provided within the manuscript or supplementary information files. The mass spectrometry proteomics data have been deposited to the ProteomeXchange Consortium via the PRIDE partner repository with the dataset identifier PXD051694.All data used in this manuscript can be found in the supporting data values file.
