# Supplementary material for: Loss of Parkinson Disease Protein 7 (PARK7) upregulates ROS and cell migration and is associated with recurrent pregnancy loss
Source: Mol Med. 2025 Dec 5;32:27. doi: 10.1186/s10020-025-01344-w (PMC12924515; doi:10.1186/s10020-025-01344-w)
Supplement: Supplementary file 1 — Supplementary Material 1. [file 10020_2025_1344_MOESM1_ESM.docx]

**Supplementary information**

**Loss of Parkinson’s Disease Protein 7 upregulates ROS and cell migration and is associated with pregnancy loss.**

Zhiqi Yang^1^, Emily Hellwich^2^, Nisha Mohd Rafiq^3^, Alvin Joselin^4^, Doo Soon Im^4^, Gaurav Kaushik^4^, Yogesh Singh^1,5^, Biserka Mulac-Jericevic^6^, Huanhuan Jiang^7^, Irene Gonzalez-Menendez^8,9^, Leticia Quintanilla-Martinez^8,10^, Sara Y. Brucker^1^, Tilman E Schäffer^2^ and Madhuri S. Salker^1^

^1^Department of Women’s Health, University of Tübingen, Germany.

^2^Institute of Applied Physics, University of Tübingen, Germany.

^3^Interfakultäres Institut für Biochemie, University of Tübingen, Germany

^4^Department of Clinical Neurosciences, Hotchkiss Brain Institute, Cumming School of Medicine, University of Calgary, Canada

^5^Institute of Medical Genetics and Applied Genomics, University of Tübingen, Germany.

^6^Department of Physiology and Immunology, Medical School, University of Rijeka, Croatia.

^7^Reproductive Medicine Center, Department of Obstetrics and Gynecology, the First Affiliated Hospital of Anhui Medical University, China.

^8^Institute of Pathology and Neuropathology, Comprehesive Cancer Center, University Hospital Tübingen, Tübingen, Germany.

^9^Core Facility Histology, Faculty of Medicine Tübingen, University Hospital Tübingen, Tübingen, Germany.

^10^Cluster of Excellence iFIT (EXC2180) "Image-guided and Functionally Instructed Tumor Therapies" University of Tübingen, Tübingen, Germany.


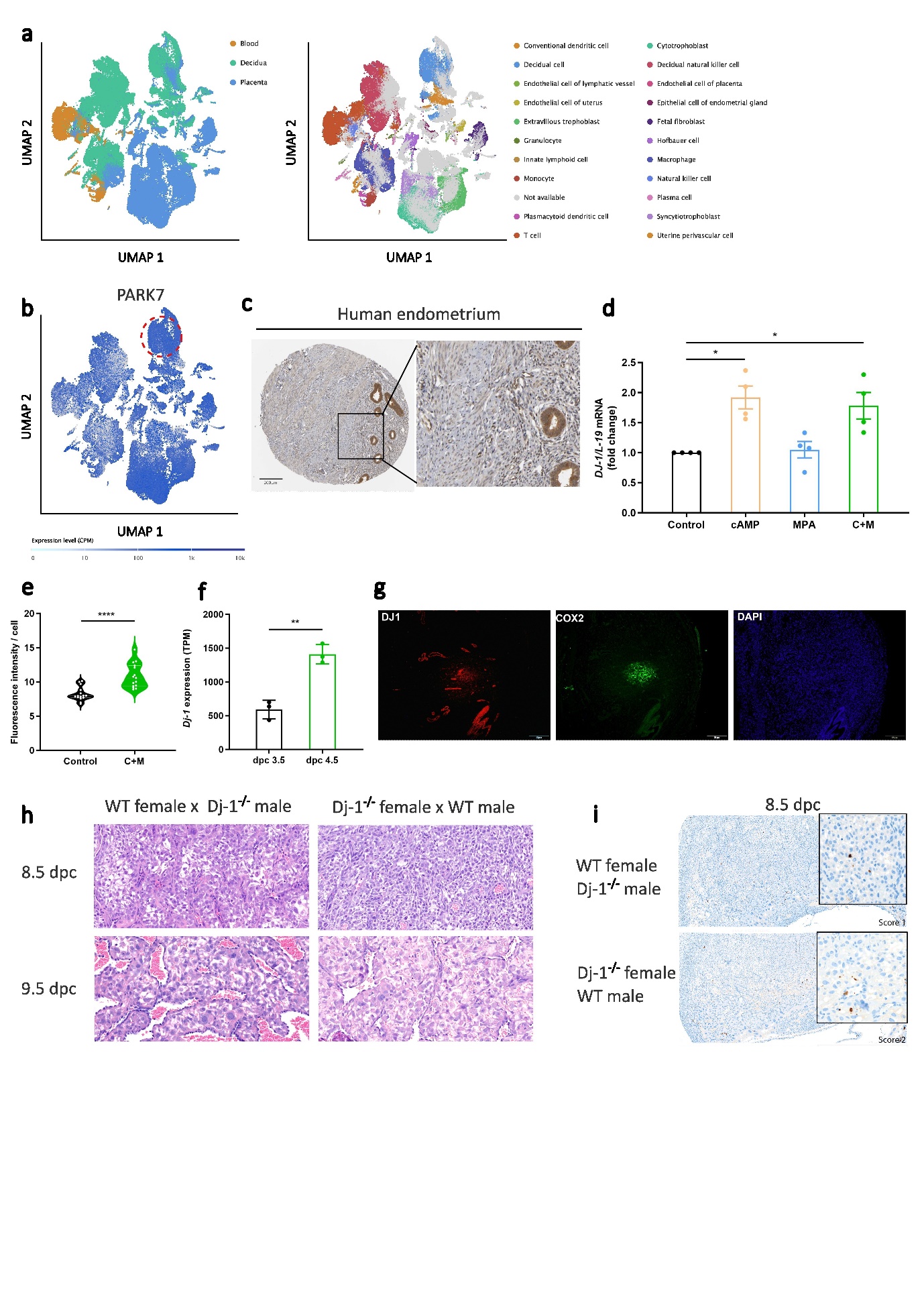


***Fig.S1*** ***Expression of PARK7/DJ-1 in human endometrium.***

***a*** *Uniform manifold approximation and projection (UMAP) clustering of tissues and cell types; Tissue compartments and cell types were annotated in the Single Cell Expression Atlas.* ***b*** *Expression of PARK7 in single cells,* *presented as counts per million (CPM), overlaid on the UMAP map. The decidual cell is indicated by a red dashed circle.* ***c*** *Representative immunohistochemistry (IHC) images of DJ-1 protein expression in human endometrium tissues. Each right panel is an enlargement of the outlined area in the left panel in its respective column in the same sample. The IHC images were downloaded from The Human Protein Atlas.* ***d*** *DJ-1 mRNA level in HESC treated with cAMP, MPA or C+M were detected by qRT-PCR (n=4). Ct values were normalized to an average Ct value of housekeeping gene L-19. The data are presented as mean ± SEM. One-way ANOVA was used to calculate statistical significance. *P < 0.05.* ***e*** *DJ-1 fluorescence intensity quantification. Quantification performed from 3 experiments with >15 cells quantified for each condition. Scale bar=200µm. Violin shows data distribution via kernel density, with median and IQR indicated by embedded box plot.* ***f*** *Mouse Dj-1 gene expression before (dpc 3.5) and during (dpc 4.5) early pregnancy (n=3) (GSE44451). The data are presented as mean ± SEM. Unpaired t-test was used to calculate statistical significance. **P < 0.01, ****P < 0.0001.* ***g*** *IF microscopy of mice uterus showing DJ-1 localization during implantation. DJ-1: Fluor 594 (red); COX2: Alexa Fluor 488 (green); nucleus: DAPI (blue).* ***h*** *Hematoxylin and eosin (H&E) staining of implantation sites at 8.5 and 9.5 dpc from wild-type (WT) females (n = 5, 8.5 dpc; n=1, 9.5 dpc) and Dj-1⁻/⁻ females (n=6, 8.5 dpc; n=2, 9.5 dpc) mated with reciprocal genotypes. Sections were analyzed to assess decidual structure and placental villi formation.* ***i*** *Immunohistochemical staining of cleaved caspase-3 in implantation sites at 8.5 dpc from wild-type (WT) females (n = 3, 8.5 dpc; n=1, 9.5 dpc) and Dj-1⁻/⁻ females (n=3, 8.5 dpc; n=2, 9.5 dpc) mated with reciprocal genotypes.*


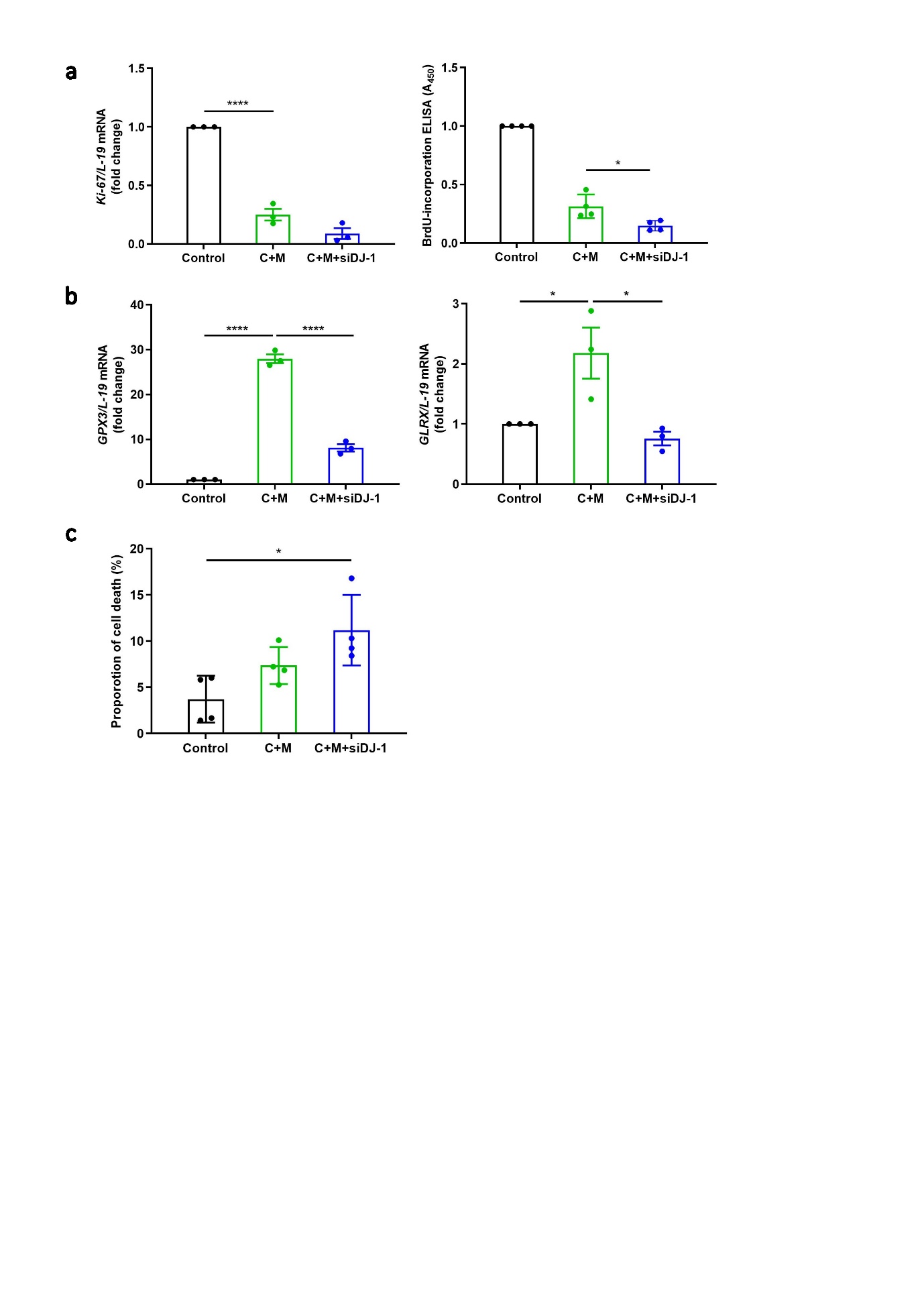


***Fig.S2 Impact of DJ-1 knockdown on cell viability and cell proliferation.***

***a*** *Ki-67 mRNA expression level in decidualizing HESCs with transfection of siDJ-1 (n=4, left);* *BrdU ELISA assay was carried out after treatment (n=4, right). Absorbance was measured at 450 nm.* ***b*** *GPX3 and GLRX mRNA expression level in decidualizing HESCs with transfection of siDJ-1 (n=3).* ***c*** *Cell death determined using Annexin V/PI staining after 6 days decidualization treatment with or without siDJ-1 transfection compared to untreated control (n=4). The data are presented as mean ± SEM. One-way ANOVA was used to calculate statistical significance. *P < 0.05, ****P < 0.0001.*


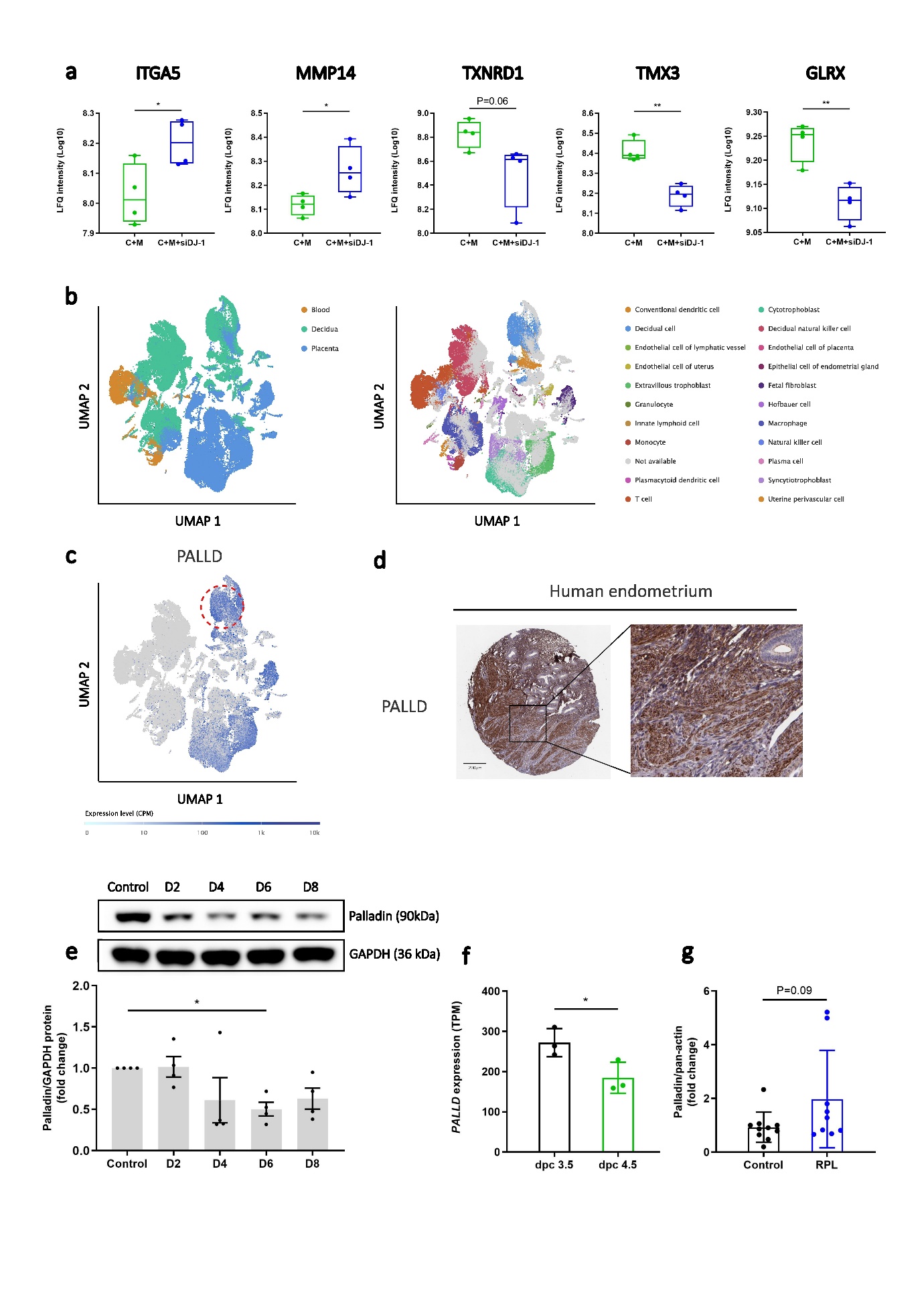


***Fig.S3 Expression of Palladin in endometrium.***

***a*** *Protein expression of ITGA5, MMP14, TXNRD1, TMX3 and GLRX in decidualized EnSC with or without siDJ-1 transfection as determined by LC/MS. Presented as LFQ intensity. Box represents the interquartile range (IQR), line indicates the median, whiskers show 1.5× IQR, unpaired t-test was used to calculate statistical significance. *P < 0.05, **P < 0.01.* ***b*** *Uniform manifold approximation and projection (UMAP) clustering of tissues and cell types; Tissue compartments and cell types were annotated in the Single Cell Expression Atlas.* ***c*** *Expression of PALLD in single cells, presented as counts per million (CPM), overlaid on the UMAP map. The decidual cell is indicated by a red dashed circle.* ***d*** *Representative immunohistochemistry (IHC) images of Palladin protein expression in human endometrium tissues. Each right panel is an enlargement of the outlined area in the left panel in its respective column in the same sample. The IHC images were downloaded from The Human Protein Atlas.* ***e*** *Western blot analysis of Palladin expression during decidualization in EnSC (n=6). GAPDH was used as a loading control.* ***f*** *Mouse PALLD gene expression before (dpc 3.5) and during (dpc 4.5) early pregnancy (n=3) (GSE44451).* ***g*** *Western blot analysis of Palladin protein expression in endometrial tissue from patients with infertility (n=10) and recurrent pregnancy loss (n=9). Pan-actin was used as a loading control. The data are presented as mean ± SEM. Unpaired t-test and one-way ANOVA was used to calculate statistical significance. *P < 0.05.*

*
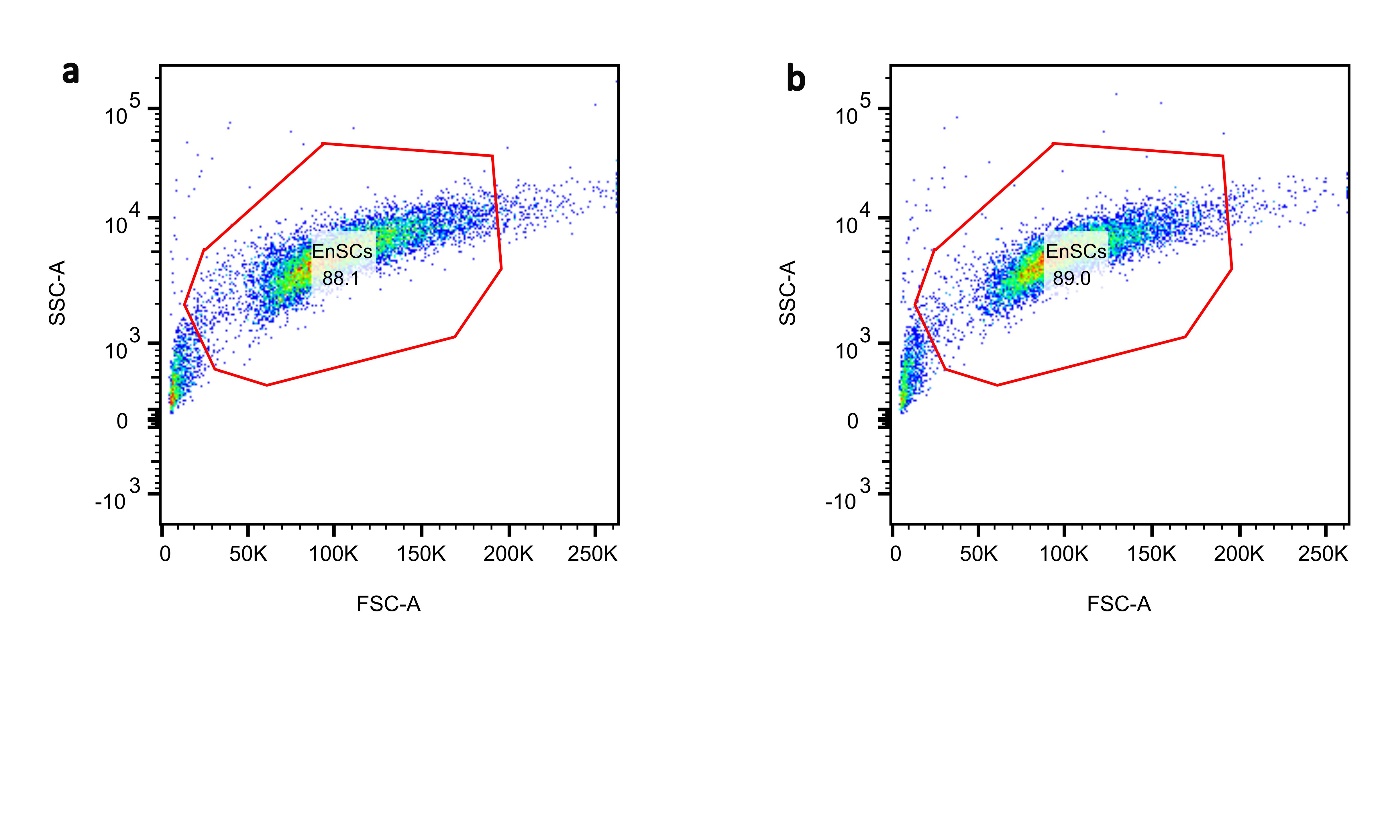
*

***Fig.S4: Gating strategy of fluorescence-activated cell sorting analysis***

***a, b*** *Gating strategy for single cells, illustrated in human endometrial stromal cell (EnSCs) population. Forward (FSC-A) and side scatter (SSC-A) are adjusted to minimize events on the axes, resulting in a single-cell population including > 80 % of total cells (****a*** *C+M,* ***b*** *C+M+siDJ-1). Each dot or point on the plot represents an individual cell that has passed through the laser. Gating strategy has been applied on EnSCs population to exclude debris, dead cells and doublets.*

***
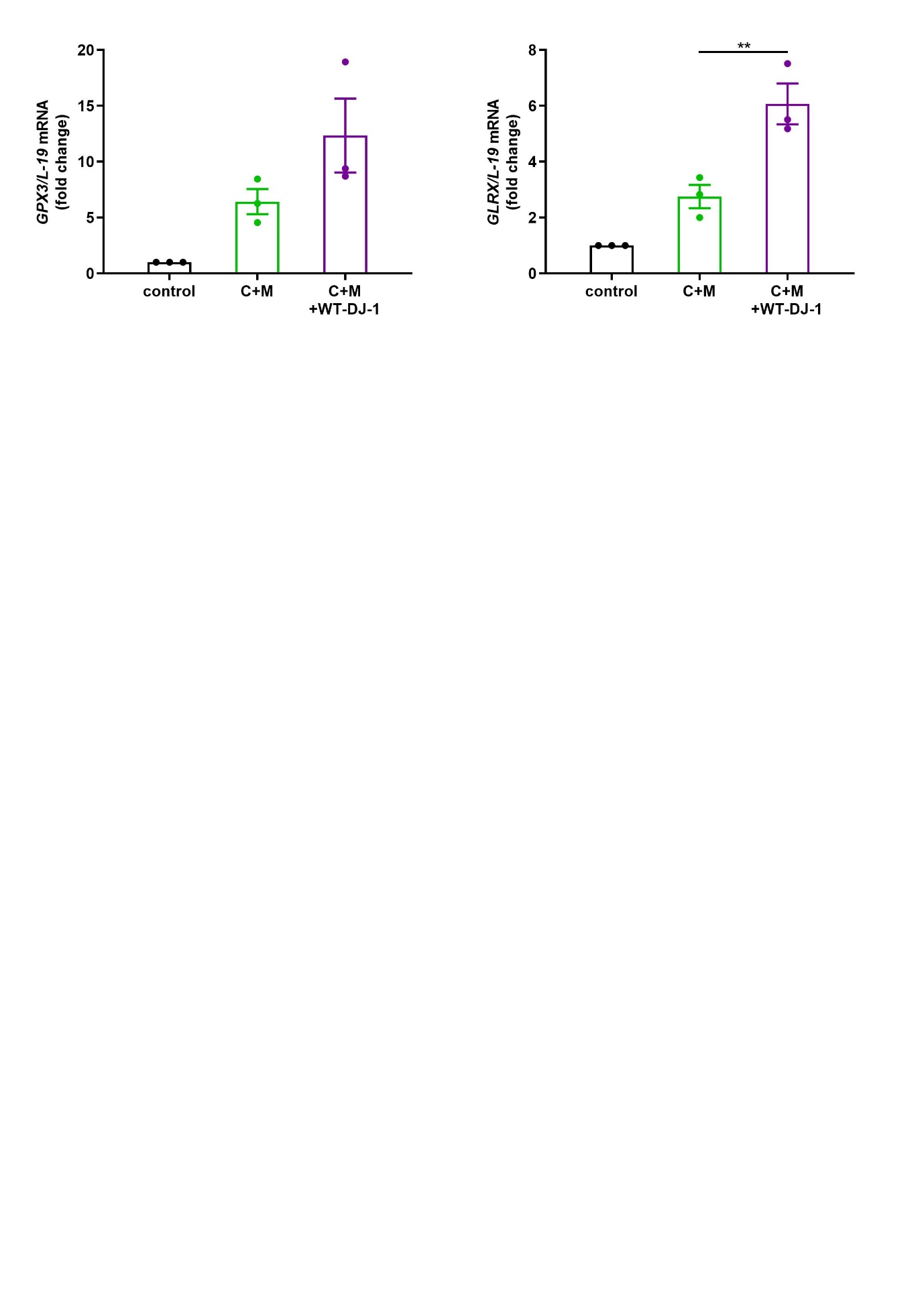
***

***Fig.S5 Overexpression of DJ-1 increase the expression of antioxidants.***

*GPX3 and GLRX mRNA expression level in decidualizing HESCs with or without transfection of wtDJ-1 (n=4)****.*** *The data are presented as mean ± SEM. One-way ANOVA was used to calculate statistical significance. **P < 0.01.*

**
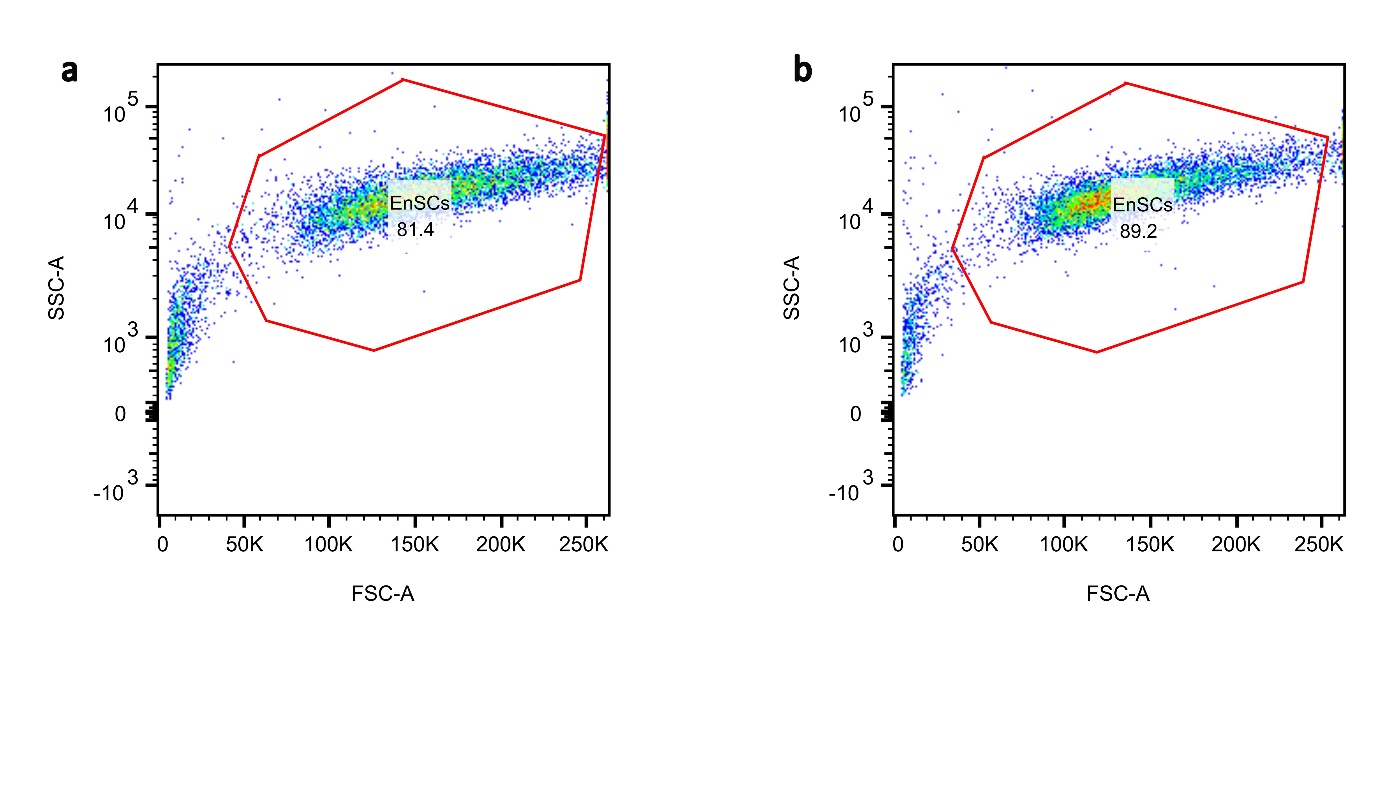
**

***Fig.S6: Gating strategy of fluorescence-activated cell sorting analysis***

*Gating strategy for single cells, illustrated in human endometrial stromal cell (EnSCs) population. Forward (FSC-A) and side scatter (SSC-A) are adjusted to minimize events on the axes, resulting in a single-cell population including > 80 % of total cells (****a*** *C+M,* ***b*** *C+M+wt-DJ-1). Each dot or point on the plot represents an individual cell that has passed through the laser. Gating strategy has been applied on EnSCs population to exclude debris, dead cells and doublets.*


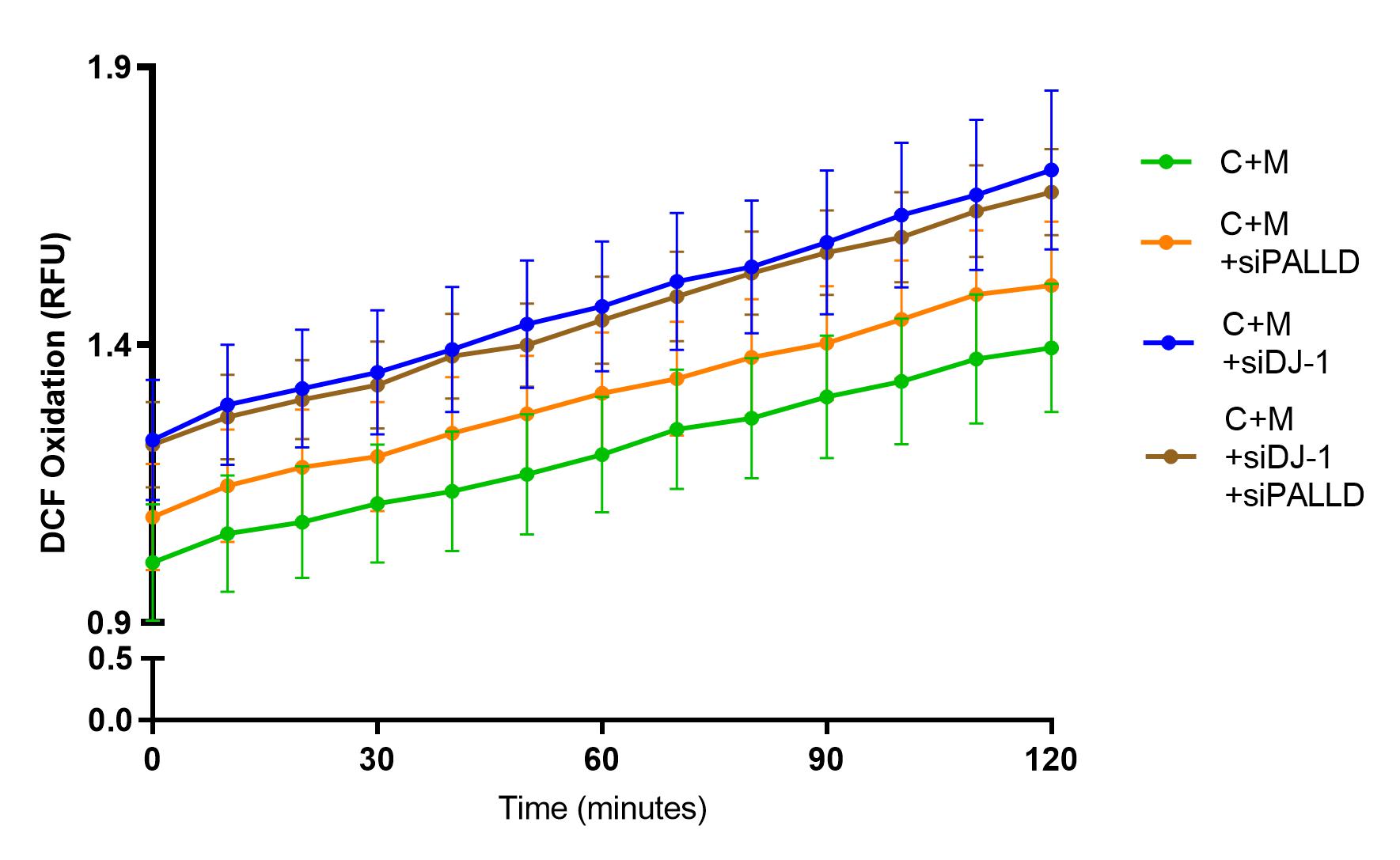


***Fig.S7 Impact of PALLD knockdown on ROS level in decidualized EnSC following the loss of DJ-1.***

*ROS DCFDA fluorescence intensity at the indicated time points (n=5). The data are presented as mean ± SEM. One-way ANOVA was used to calculate statistical significance.*


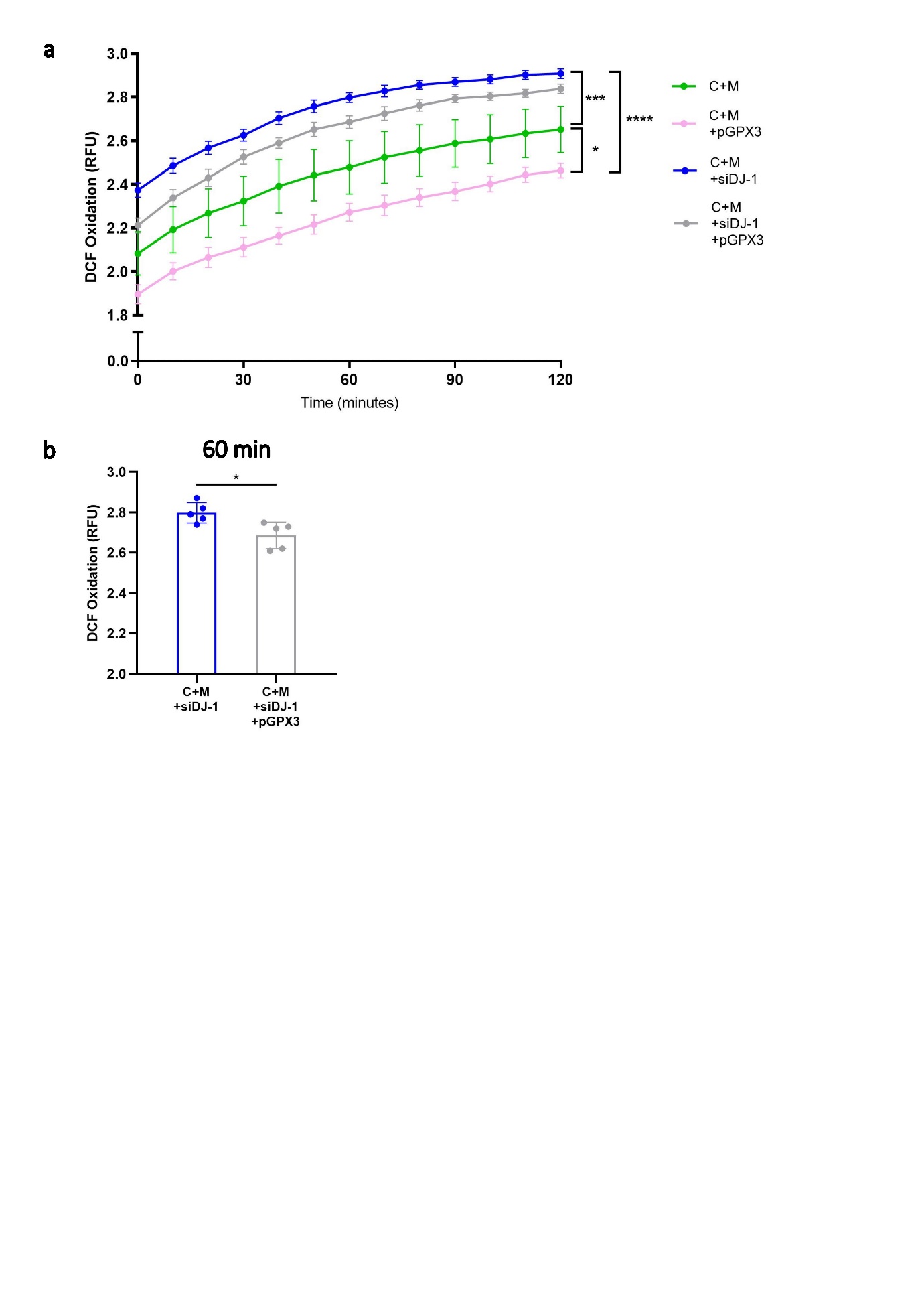


***Fig.S8 Impact of GPX3 overexpression on DJ-1 knockdown EnSC.***

***a*** *ROS DCFDA fluorescence intensity at the indicated time points (n=5). The data are presented as mean ± SEM. One-way ANOVA was used to calculate statistical significance. *P < 0.05, ***P < 0.001, ****P < 0.0001.* ***b*** *ROS DCFDA fluorescence intensity at 60 min (n=5). The data are presented as mean ± SEM. Unpaired t-test was used to calculate statistical significance. *P < 0.05.*
