# Supplementary material for: Loss of Parkinson Disease Protein 7 (PARK7) upregulates ROS and cell migration and is associated with recurrent pregnancy loss
Source: Mol Med. 2025 Dec 5;32:27. doi: 10.1186/s10020-025-01344-w (PMC12924515; doi:10.1186/s10020-025-01344-w)
Supplement: Supplementary file 3 — Supplementary Material 3. [file 10020_2025_1344_MOESM3_ESM.pptx]

## Slide 1
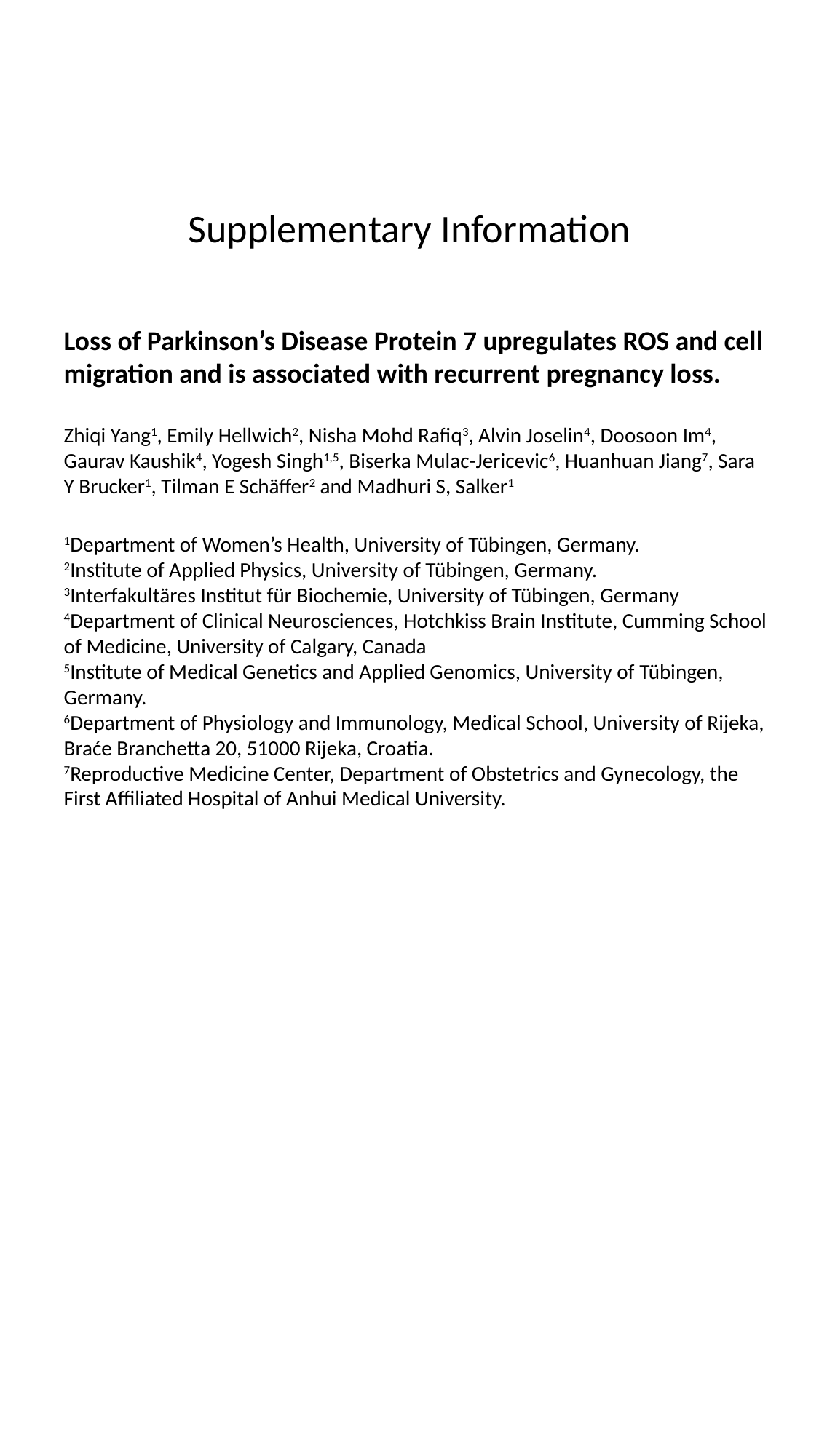

Supplementary Information
Loss of Parkinson’s Disease Protein 7 upregulates ROS and cell migration and is associated with recurrent pregnancy loss.
Zhiqi Yang1, Emily Hellwich2, Nisha Mohd Rafiq3, Alvin Joselin4, Doosoon Im4, Gaurav Kaushik4, Yogesh Singh1,5, Biserka Mulac-Jericevic6, Huanhuan Jiang7, Sara Y Brucker1, Tilman E Schäffer2 and Madhuri S, Salker1
1Department of Women’s Health, University of Tübingen, Germany.
2Institute of Applied Physics, University of Tübingen, Germany.
3Interfakultäres Institut für Biochemie, University of Tübingen, Germany
4Department of Clinical Neurosciences, Hotchkiss Brain Institute, Cumming School of Medicine, University of Calgary, Canada
5Institute of Medical Genetics and Applied Genomics, University of Tübingen, Germany.
6Department of Physiology and Immunology, Medical School, University of Rijeka, Braće Branchetta 20, 51000 Rijeka, Croatia.
7Reproductive Medicine Center, Department of Obstetrics and Gynecology, the First Affiliated Hospital of Anhui Medical University.

## Slide 2
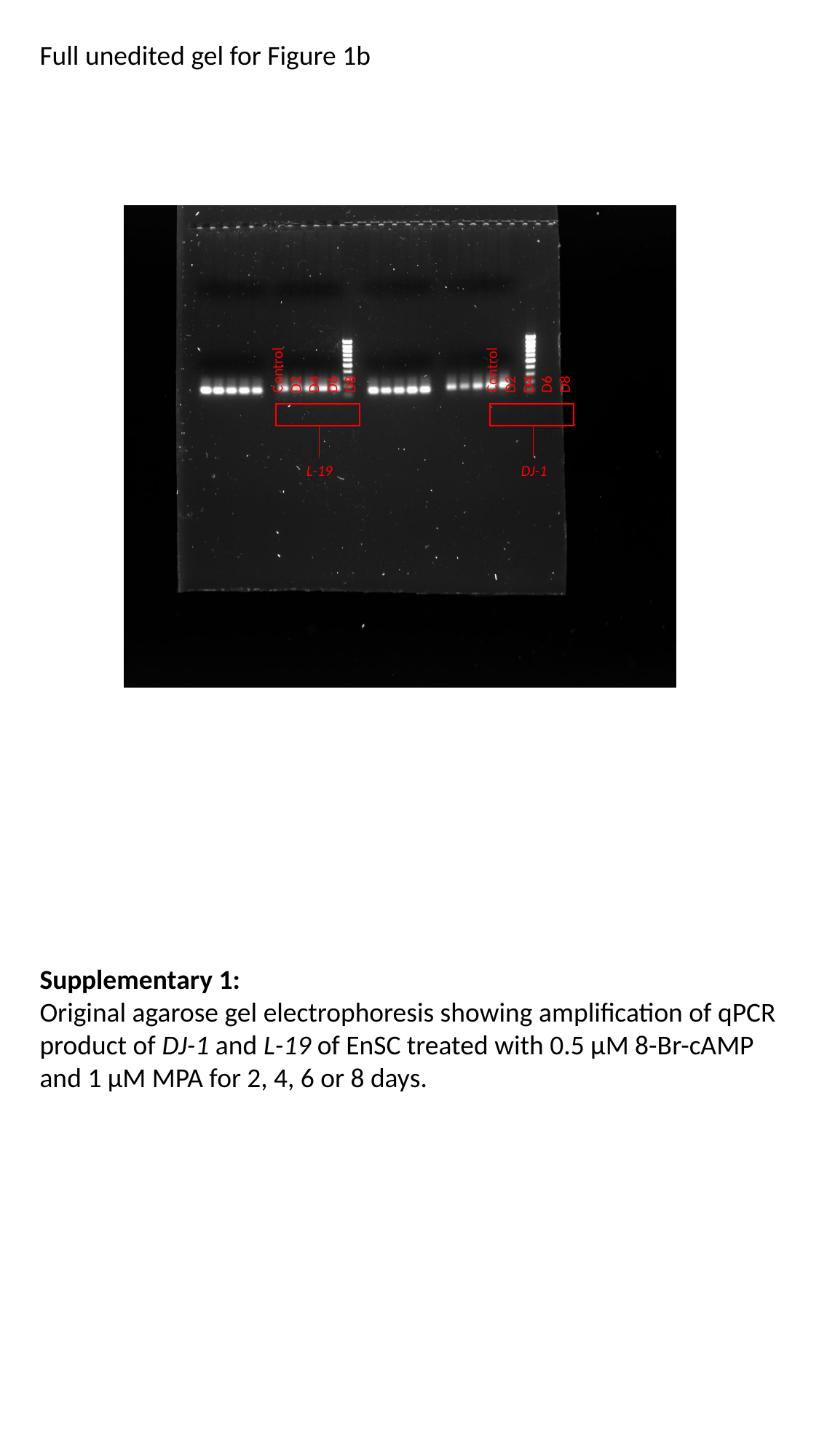

Full unedited gel for Figure 1b
Control
D2
D4
D6
D8
Control
D2
D4
D6
D8
L-19
DJ-1
Supplementary 1:
Original agarose gel electrophoresis showing amplification of qPCR product of DJ-1 and L-19 of EnSC treated with 0.5 μM 8-Br-cAMP and 1 μM MPA for 2, 4, 6 or 8 days.

## Slide 3
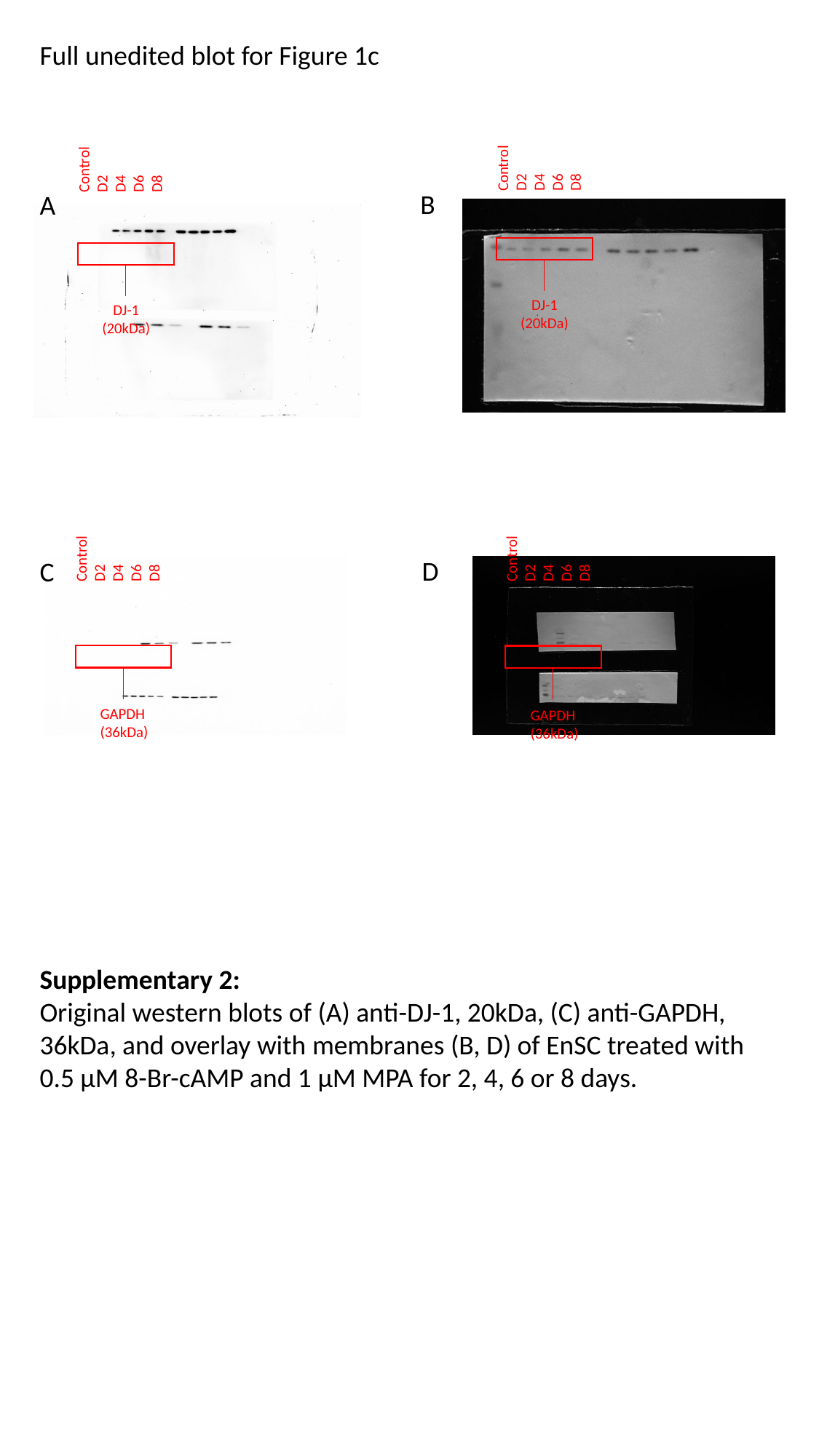

Full unedited blot for Figure 1c
Control
D2
D4
D6
D8
Control
D2
D4
D6
D8
B
A
DJ-1
(20kDa)
DJ-1
(20kDa)
Control
D2
D4
D6
D8
Control
D2
D4
D6
D8
D
C
GAPDH (36kDa)
GAPDH (36kDa)
Supplementary 2:
Original western blots of (A) anti-DJ-1, 20kDa, (C) anti-GAPDH, 36kDa, and overlay with membranes (B, D) of EnSC treated with 0.5 μM 8-Br-cAMP and 1 μM MPA for 2, 4, 6 or 8 days.

## Slide 4
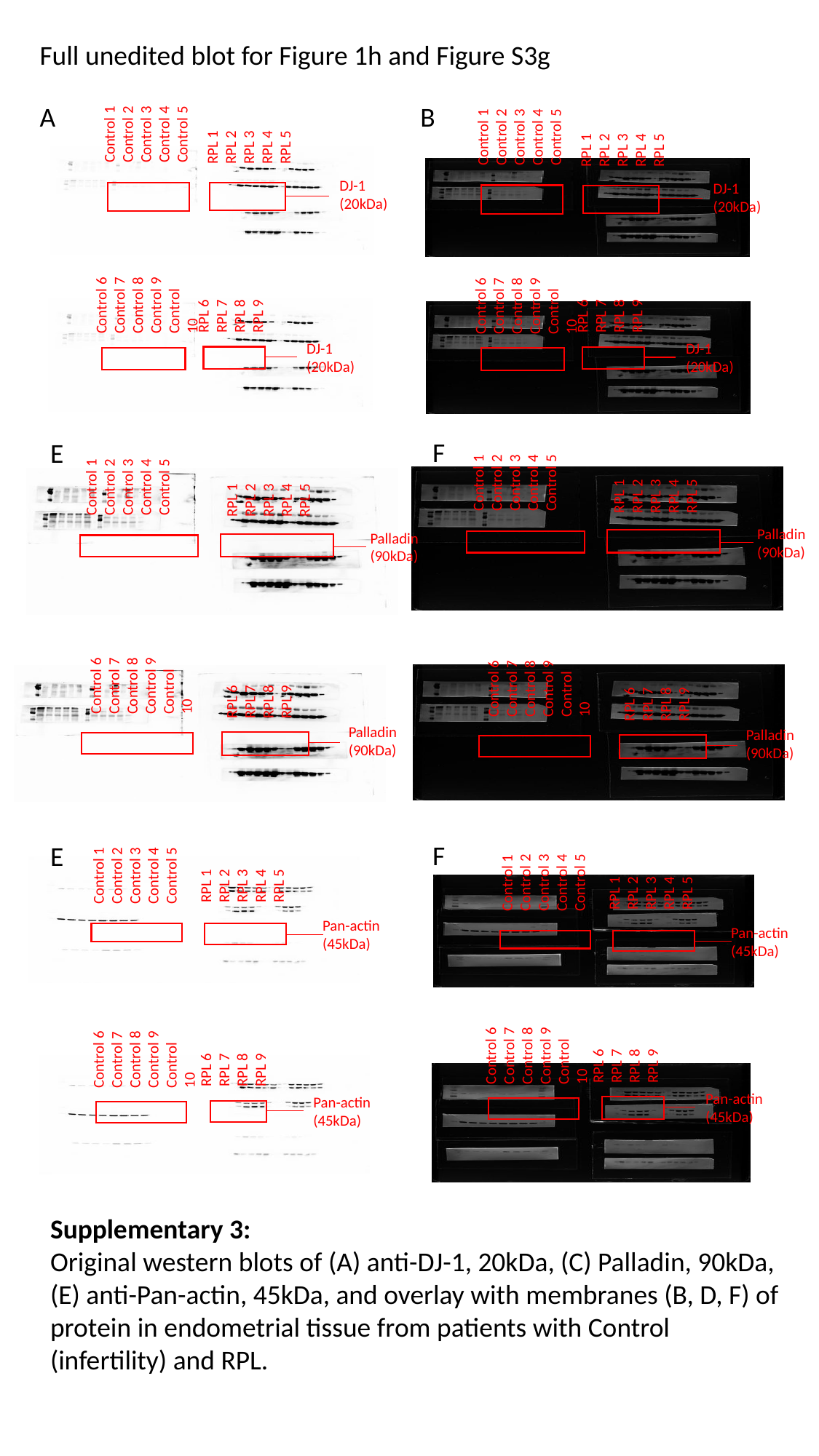

Full unedited blot for Figure 1h and Figure S3g
Control 1
Control 2
Control 3
Control 4
Control 5
RPL 1
RPL 2
RPL 3
RPL 4
RPL 5
Control 1
Control 2
Control 3
Control 4
Control 5
RPL 1
RPL 2
RPL 3
RPL 4
RPL 5
B
A
DJ-1
(20kDa)
DJ-1
(20kDa)
RPL 6
RPL 7
RPL 8
RPL 9
RPL 6
RPL 7
RPL 8
RPL 9
Control 6
Control 7
Control 8
Control 9
Control 10
Control 6
Control 7
Control 8
Control 9
Control 10
DJ-1
(20kDa)
DJ-1
(20kDa)
F
E
Control 1
Control 2
Control 3
Control 4
Control 5
RPL 1
RPL 2
RPL 3
RPL 4
RPL 5
Control 1
Control 2
Control 3
Control 4
Control 5
RPL 1
RPL 2
RPL 3
RPL 4
RPL 5
Palladin
(90kDa)
Palladin
(90kDa)
Control 6
Control 7
Control 8
Control 9
Control 10
Control 6
Control 7
Control 8
Control 9
Control 10
RPL 6
RPL 7
RPL 8
RPL 9
RPL 6
RPL 7
RPL 8
RPL 9
Palladin
(90kDa)
Palladin
(90kDa)
RPL 1
RPL 2
RPL 3
RPL 4
RPL 5
Control 1
Control 2
Control 3
Control 4
Control 5
F
E
RPL 1
RPL 2
RPL 3
RPL 4
RPL 5
Control 1
Control 2
Control 3
Control 4
Control 5
Pan-actin
(45kDa)
Pan-actin
(45kDa)
RPL 6
RPL 7
RPL 8
RPL 9
Control 6
Control 7
Control 8
Control 9
Control 10
RPL 6
RPL 7
RPL 8
RPL 9
Control 6
Control 7
Control 8
Control 9
Control 10
Pan-actin
(45kDa)
Pan-actin
(45kDa)
Supplementary 3:
Original western blots of (A) anti-DJ-1, 20kDa, (C) Palladin, 90kDa, (E) anti-Pan-actin, 45kDa, and overlay with membranes (B, D, F) of protein in endometrial tissue from patients with Control (infertility) and RPL.

## Slide 5
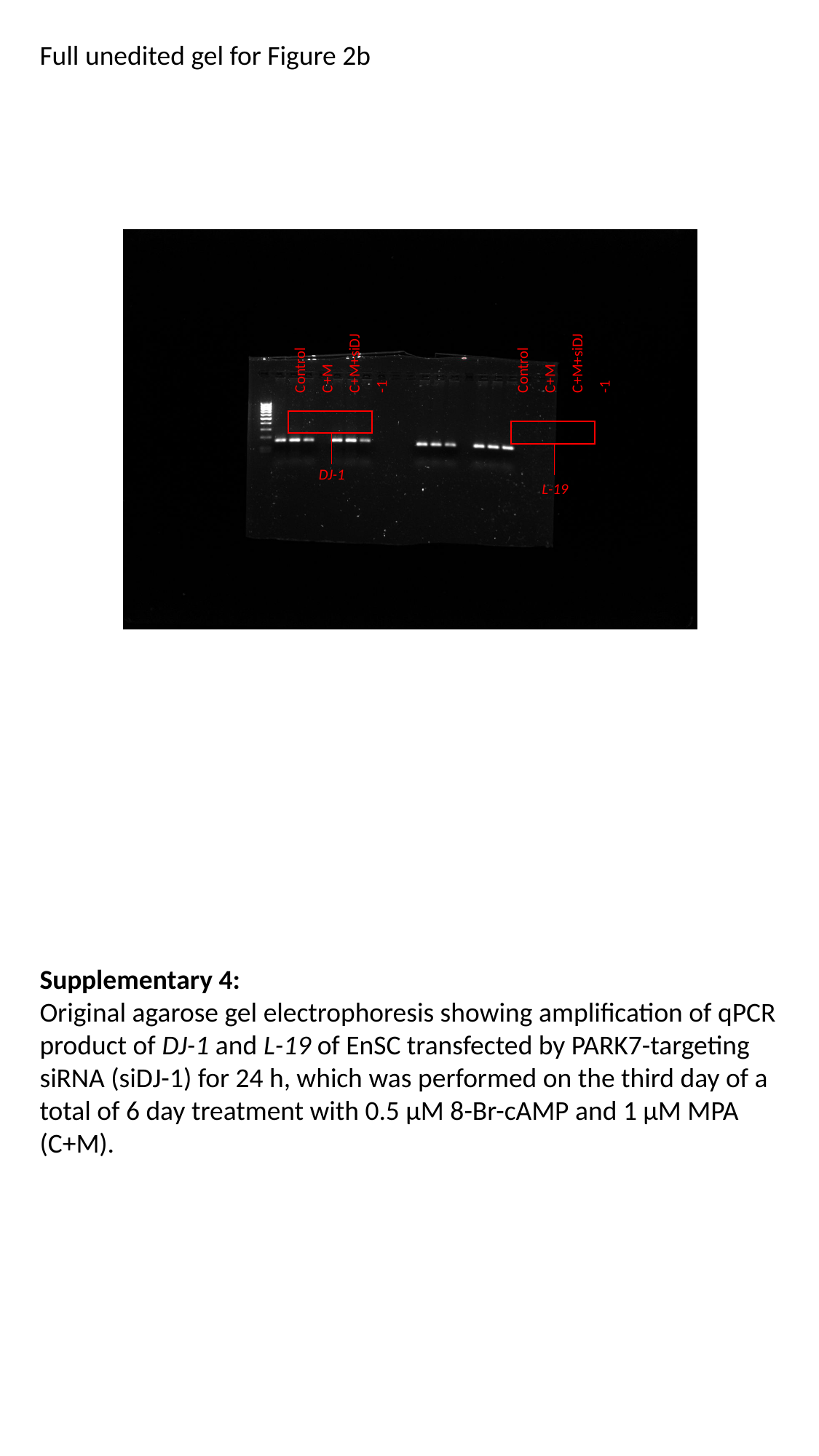

Full unedited gel for Figure 2b
Control
C+M
C+M+siDJ-1
Control
C+M
C+M+siDJ-1
DJ-1
L-19
Supplementary 4:
Original agarose gel electrophoresis showing amplification of qPCR product of DJ-1 and L-19 of EnSC transfected by PARK7-targeting siRNA (siDJ-1) for 24 h, which was performed on the third day of a total of 6 day treatment with 0.5 μM 8-Br-cAMP and 1 μM MPA (C+M).

## Slide 6
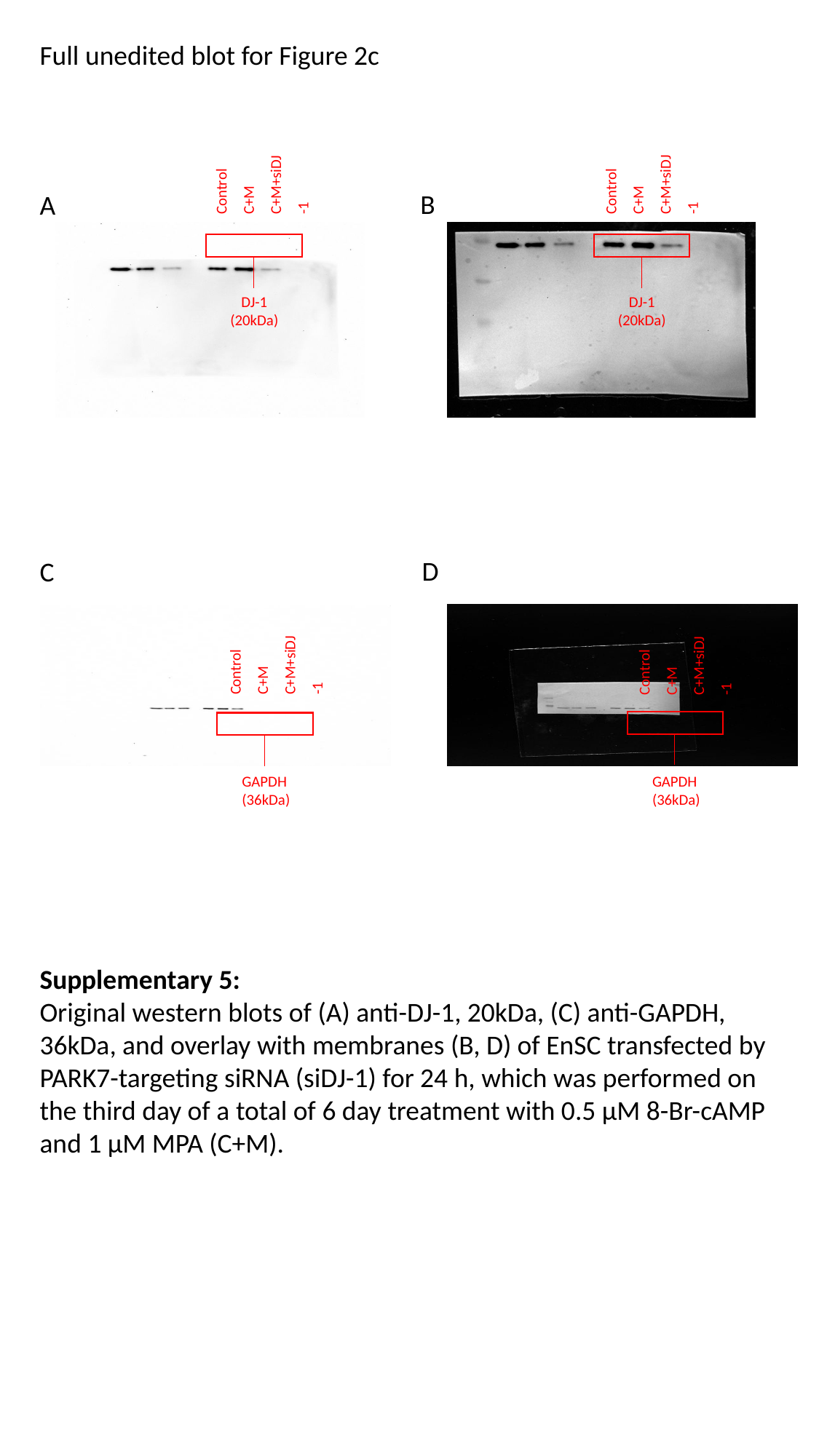

Full unedited blot for Figure 2c
Control
C+M
C+M+siDJ-1
Control
C+M
C+M+siDJ-1
B
A
DJ-1
(20kDa)
DJ-1
(20kDa)
D
C
Control
C+M
C+M+siDJ-1
Control
C+M
C+M+siDJ-1
GAPDH (36kDa)
GAPDH (36kDa)
Supplementary 5:
Original western blots of (A) anti-DJ-1, 20kDa, (C) anti-GAPDH, 36kDa, and overlay with membranes (B, D) of EnSC transfected by PARK7-targeting siRNA (siDJ-1) for 24 h, which was performed on the third day of a total of 6 day treatment with 0.5 μM 8-Br-cAMP and 1 μM MPA (C+M).

## Slide 7
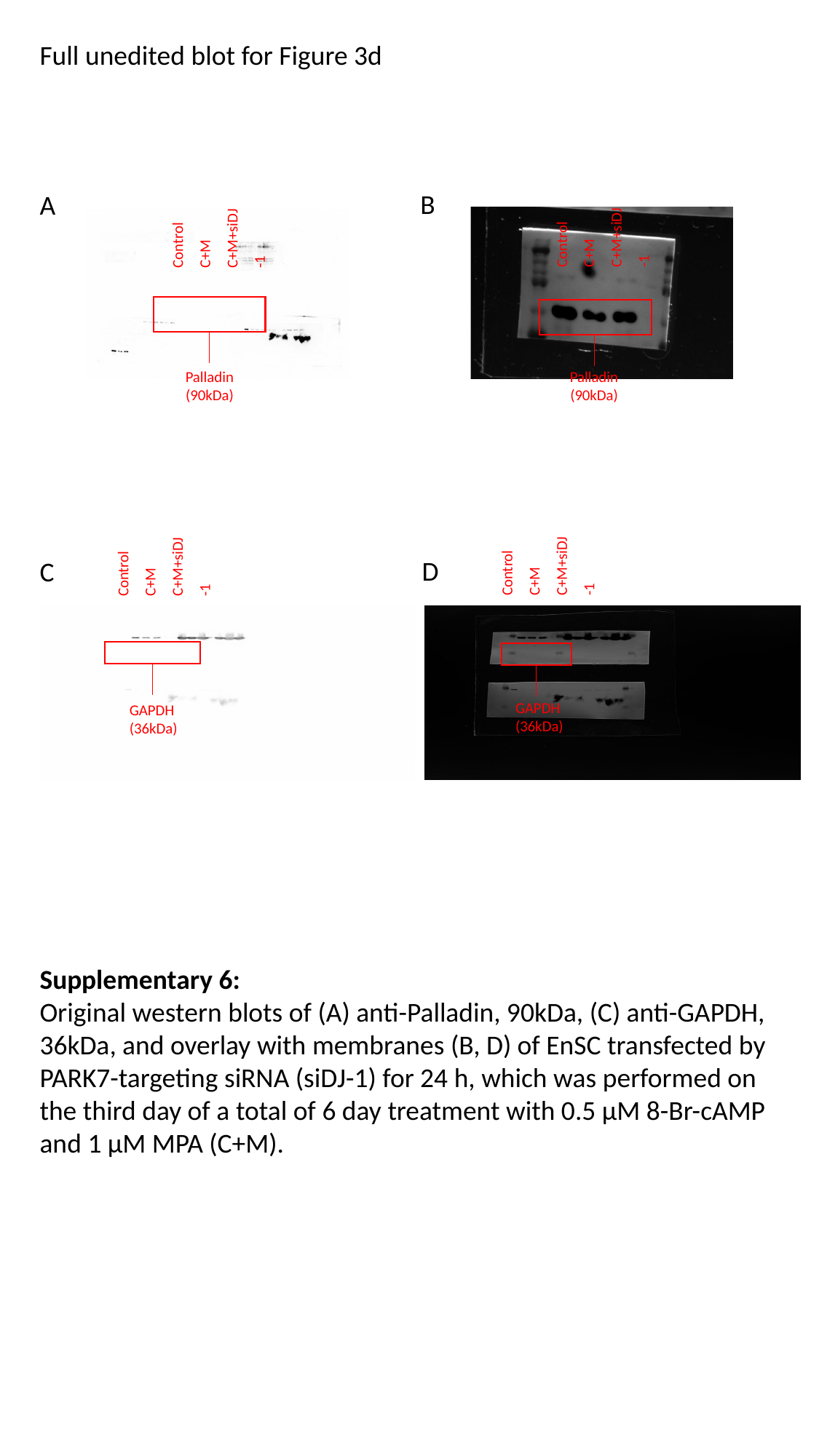

Full unedited blot for Figure 3d
B
A
Control
C+M
C+M+siDJ-1
Control
C+M
C+M+siDJ-1
Palladin
(90kDa)
Palladin
(90kDa)
Control
C+M
C+M+siDJ-1
Control
C+M
C+M+siDJ-1
D
C
GAPDH (36kDa)
GAPDH (36kDa)
Supplementary 6:
Original western blots of (A) anti-Palladin, 90kDa, (C) anti-GAPDH, 36kDa, and overlay with membranes (B, D) of EnSC transfected by PARK7-targeting siRNA (siDJ-1) for 24 h, which was performed on the third day of a total of 6 day treatment with 0.5 μM 8-Br-cAMP and 1 μM MPA (C+M).

## Slide 8
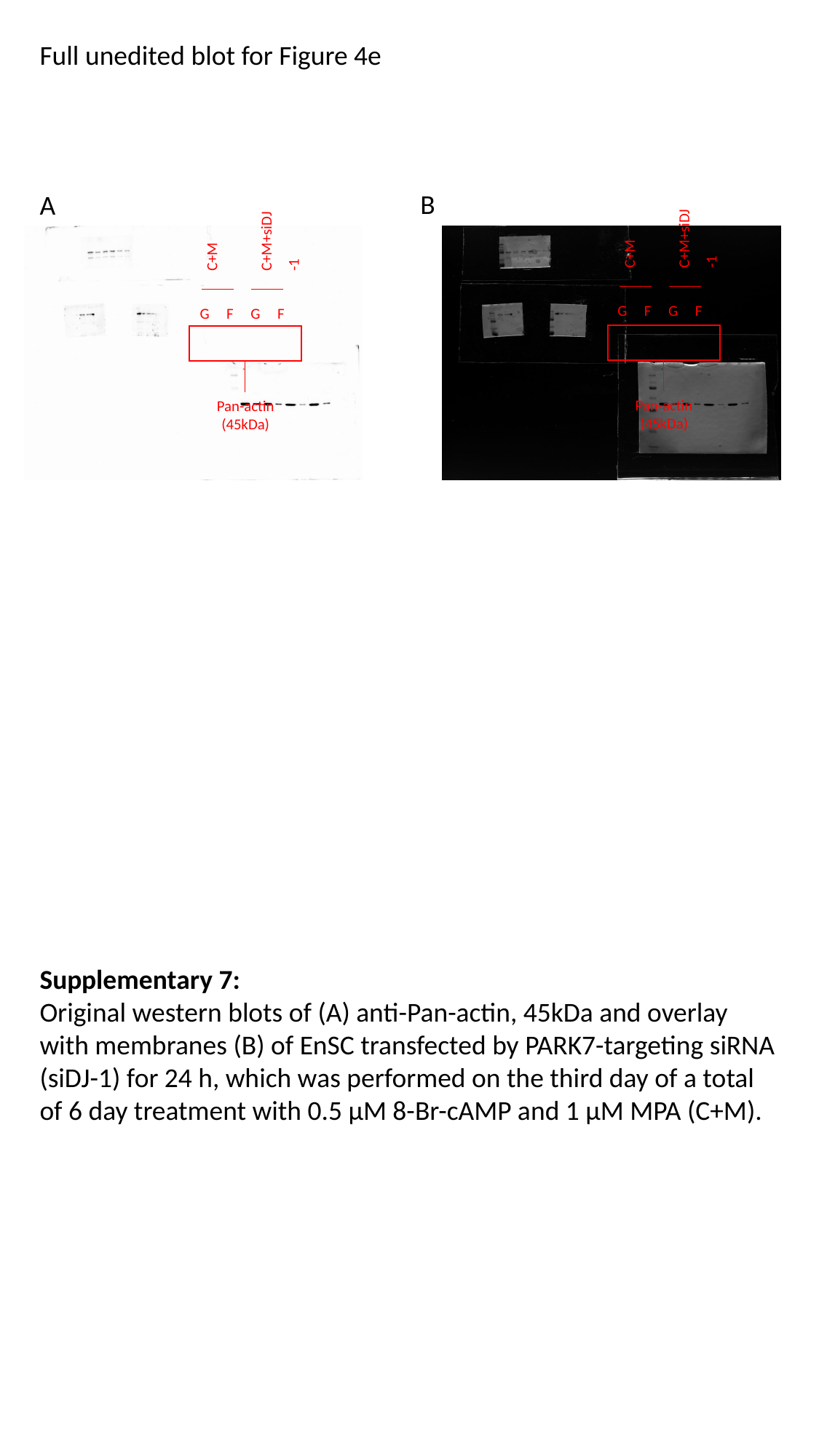

Full unedited blot for Figure 4e
B
A
C+M
C+M+siDJ-1
C+M
C+M+siDJ-1
G F G F
G F G F
Pan-actin
(45kDa)
Pan-actin
(45kDa)
Supplementary 7:
Original western blots of (A) anti-Pan-actin, 45kDa and overlay with membranes (B) of EnSC transfected by PARK7-targeting siRNA (siDJ-1) for 24 h, which was performed on the third day of a total of 6 day treatment with 0.5 μM 8-Br-cAMP and 1 μM MPA (C+M).

## Slide 9
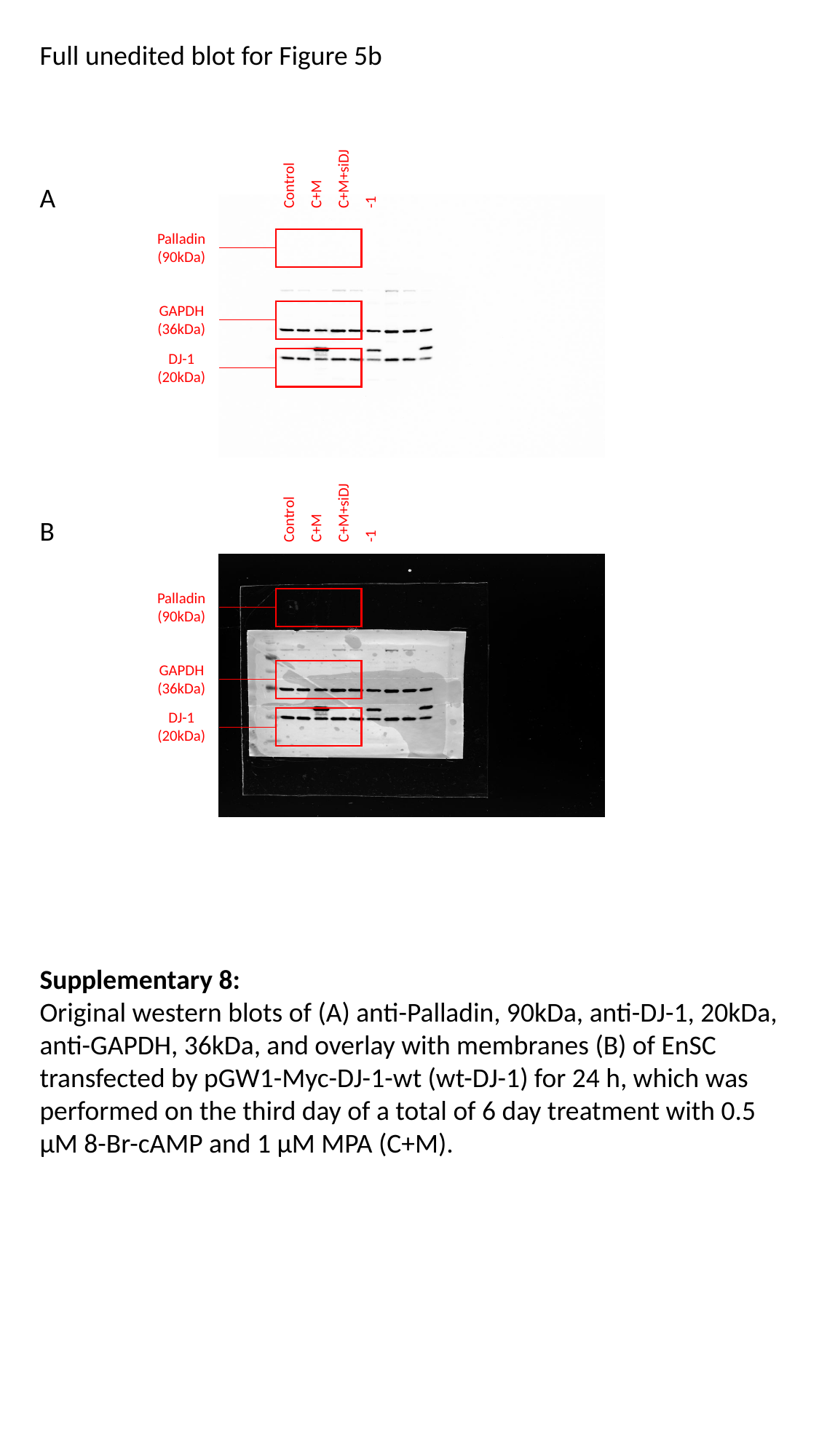

Full unedited blot for Figure 5b
Control
C+M
C+M+siDJ-1
A
Palladin
(90kDa)
GAPDH
(36kDa)
DJ-1
(20kDa)
Control
C+M
C+M+siDJ-1
B
Palladin
(90kDa)
GAPDH
(36kDa)
DJ-1
(20kDa)
Supplementary 8:
Original western blots of (A) anti-Palladin, 90kDa, anti-DJ-1, 20kDa, anti-GAPDH, 36kDa, and overlay with membranes (B) of EnSC transfected by pGW1-Myc-DJ-1-wt (wt-DJ-1) for 24 h, which was performed on the third day of a total of 6 day treatment with 0.5 μM 8-Br-cAMP and 1 μM MPA (C+M).

## Slide 10
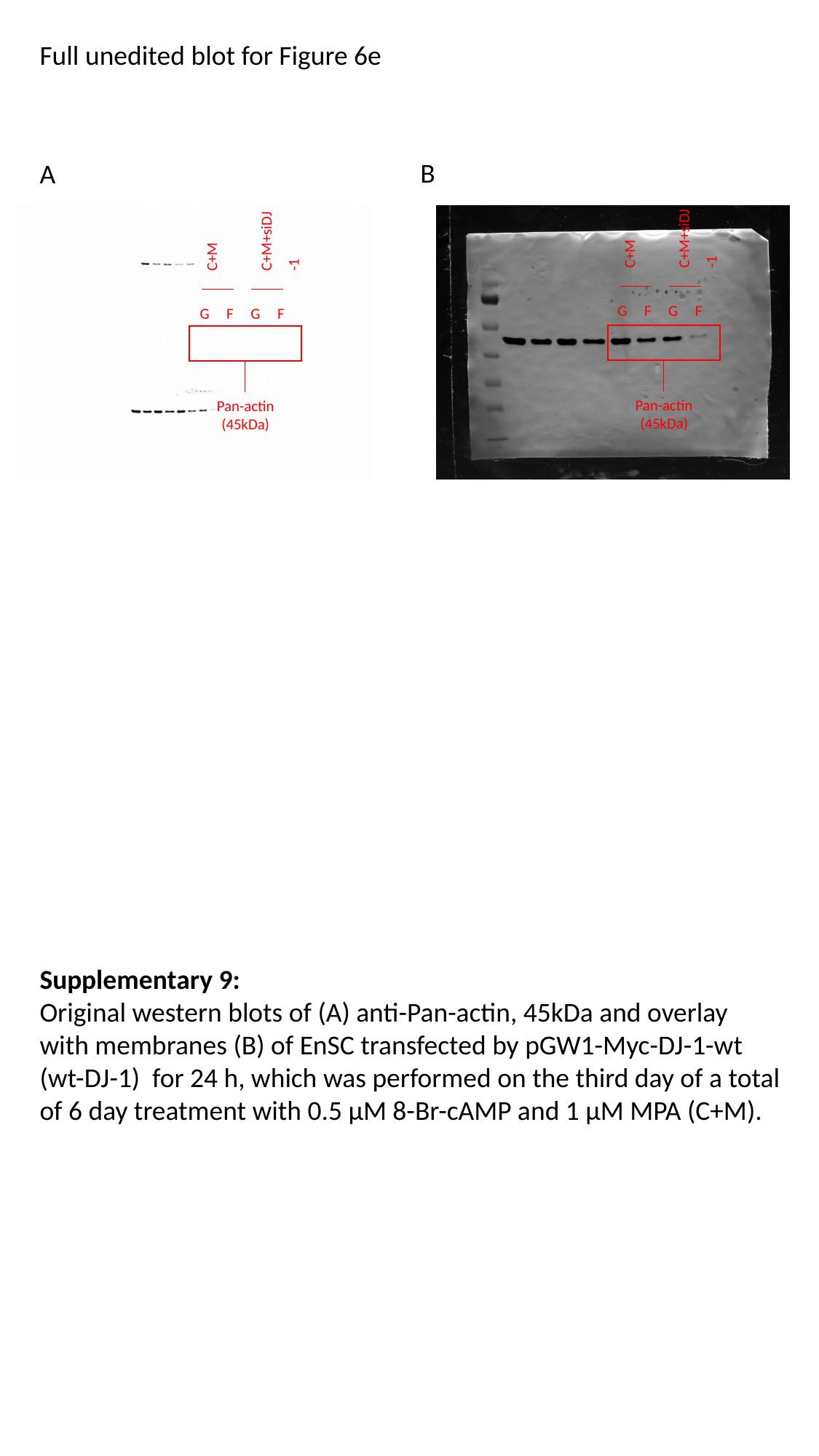

Full unedited blot for Figure 6e
B
A
C+M
C+M+siDJ-1
C+M
C+M+siDJ-1
G F G F
G F G F
Pan-actin
(45kDa)
Pan-actin
(45kDa)
Supplementary 9:
Original western blots of (A) anti-Pan-actin, 45kDa and overlay with membranes (B) of EnSC transfected by pGW1-Myc-DJ-1-wt (wt-DJ-1) for 24 h, which was performed on the third day of a total of 6 day treatment with 0.5 μM 8-Br-cAMP and 1 μM MPA (C+M).
